# Dual Targeting of CXCR4 and CXCL12 by 1,2,4-Triazole Derivatives: A Computational Approach Against Lung Cancer Metastasis

**DOI:** 10.5812/ijpr-171955

**Published:** 2026-08-18

**Authors:** Yeni Yeni, Fransiska Kurniawan, Benny Permana, Daryono Hadi Tjahjono

**Affiliations:** 1 Department of Pharmacochemistry, School of Pharmacy, Bandung Institute of Technology, Bandung, Indonesia; 2 Department of Pharmacy, Faculty of Pharmacy and Science, Universitas Muhammadiyah Prof. DR. HAMKA, Jakarta, Indonesia

**Keywords:** Anti-metastatic, CXCL12, CXCR4, Lung Cancer, 1,2,4-triazoles

## Abstract

**Background:**

Migration of lung cancer cells to distant sites is largely mediated by the CXCR4/CXCL12 axis. Meanwhile, 1,2,4-triazole derivatives have shown antiproliferative activity in lung cancer and have been reported to inhibit metastasis in pancreatic and breast cancer.

**Objectives:**

This study aimed to screen 1,2,4-triazole derivatives with high potential to target the CXCR4/CXCL12 axis associated with lung cancer metastasis using multiple computational methods.

**Methods:**

A total of 79 1,2,4-triazole derivatives were screened using ligand-based pharmacophore modeling (LigandScout Essential 4.4.9) and molecular docking (AutoDock 4.2.6). Molecular dynamics (MD) simulations were then performed using GROMACS 2021.4 to further characterize protein–ligand interactions. Finally, compound safety, biological behavior, and drug-development feasibility were evaluated using toxicity prediction (ProTox 3.0) and pharmacokinetic and drug-likeness assessments (pkCSM).

**Results:**

Sixteen derivatives (D41-D51 and D53-D57) matched the CXCR4 and CXCL12 pharmacophore models. The CXCR4 model (model 8) comprised two hydrophobic features, one aromatic ring, and one hydrogen bond donor, whereas the CXCL12 model (model 3) included one hydrophobic feature, two aromatic rings, and three hydrogen bond acceptors. Docking studies against CXCR4 and CXCL12 indicated that D54 and D57 had more favorable predicted docking scores than mavorixafor (a CXCR4 inhibitor) and LIT-927 (a CXCL12 inhibitor). These findings were supported by MD simulations, as indicated by stable root mean square deviation (RMSD), root mean square fluctuation (RMSF), radius of gyration (Rg), solvent-accessible surface area (SASA), hydrogen bond (H-bond) occupancy, and Molecular Mechanics Poisson-Boltzmann Surface Area (MM-PBSA) results. Toxicity and pharmacokinetic predictions suggested that D54 and D57 had both favorable properties and potential liabilities.

**Conclusions:**

Compounds D54 and D57 were computationally prioritized as promising 1,2,4-triazole derivatives targeting the CXCR4/CXCL12 axis for further investigation in lung cancer metastasis. However, their predicted toxicity, pharmacokinetic, and drug-likeness profiles revealed several limitations that warrant further optimization and experimental validation.

## 1. Background

Lung cancer is the leading cause of cancer-associated mortality worldwide (1). The high mortality rate is primarily driven by metastatic progression, which is associated with poor prognosis and therapeutic resistance (2). The CXCR4 chemokine receptor and its ligand CXCL12 play critical roles in lung cancer metastasis. Lung cancer cells expressing CXCR4 migrate to organs or tissues with high levels of CXCL12, including the lymph nodes, contralateral lung, liver, brain, and bone marrow (3). Therefore, incorporating pharmaceuticals that target the CXCR4/CXCL12 axis into treatment protocols may provide an effective approach to treating metastatic cancer.

**Figure 1.**
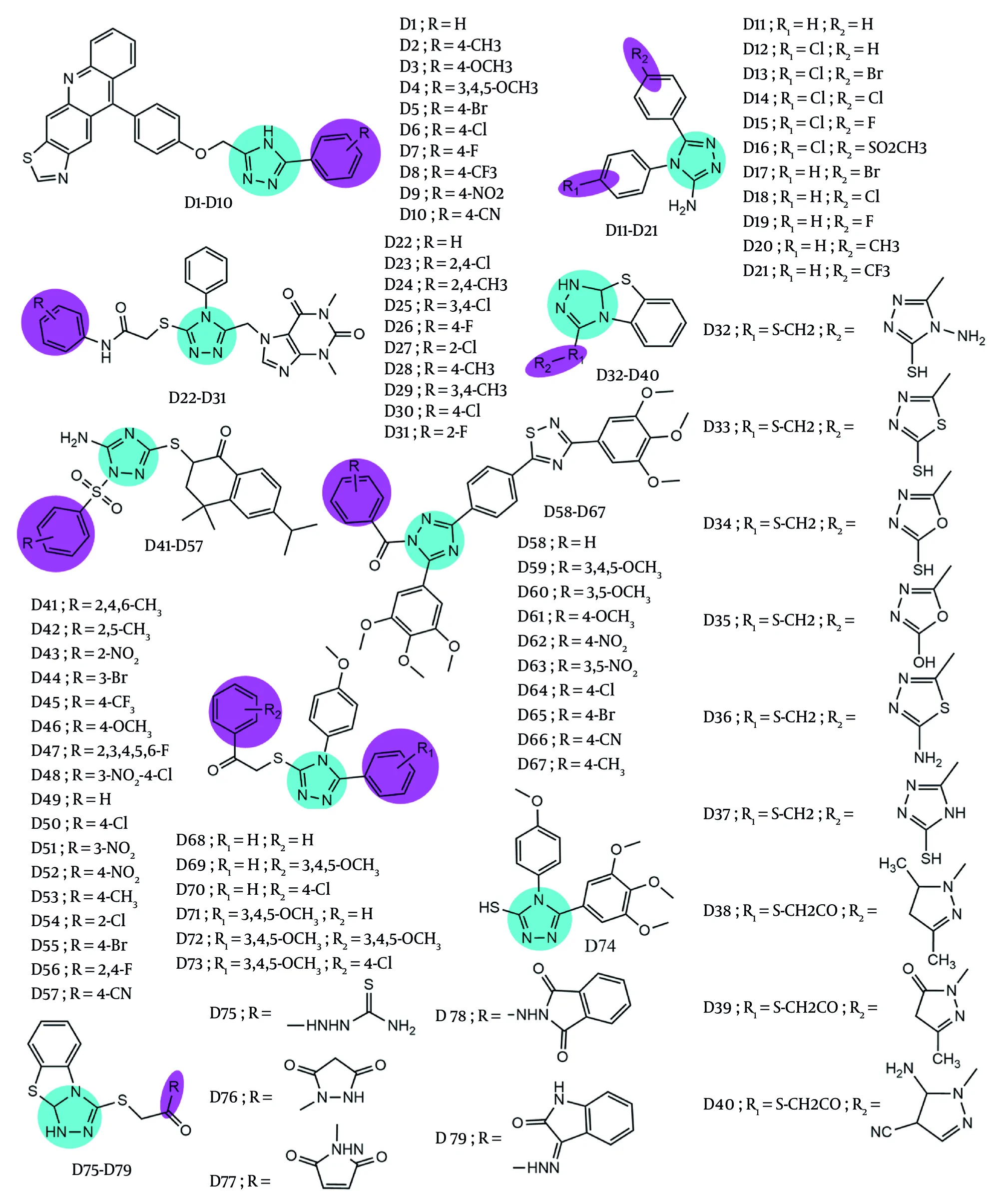
The 1,2,4-triazole derivatives (D1 - D79) as test compounds

Several 1,2,4-triazole derivatives have been investigated in vitro for antiproliferative activity against the A549 lung cancer cell line using the MTT (3-[4,5-dimethylthiazol-2-yl]-2,5-diphenyl tetrazolium bromide) assay (Figure 1) (4-11). In addition, several derivatives have reportedly demonstrated strong anti-metastatic activity in pancreatic and breast cancer (12). Computational technologies can enhance drug discovery by streamlining compound selection and increasing success rates (13).

## 2. Objectives

The present study aimed to identify 1,2,4-triazole derivatives as potential anti-metastatic candidates for lung cancer with fewer adverse effects.

## 3. Methods

### 3.1. Data Preparation

The Protein Data Bank (PDB) provided the 3D structures of CXCR4 (PDB code: 3ODU) (14) and CXCL12 (PDB code: 4UAI) (15). The 3D structures of mavorixafor (CID: 11256587) and LIT-927 (CID: 137287575) were retrieved from PubChem and used as reference compounds for the CXCR4 and CXCL12 targets, respectively. The 2D structures of 79 test compounds (Figure 1) were generated using MarvinSketch 22.8 and pre-optimized with Avogadro. The corresponding SMILES strings are provided in Appendix 1 in the Supplementary File. During pre-optimization, the Auto Optimize tool was used to optimize molecular geometry using molecular mechanics. In Avogadro, the UFF (Universal Force Field) was selected, with 4 steps per update and the steepest descent algorithm.

### 3.2. Ligand-Based Pharmacophore Modeling

Pharmacophore models for CXCR4 and CXCL12 were generated using LigandScout Essential 4.4.9 (16). For each target, the 50 most active compounds were retrieved from BindingDB (17). CXCR4 ligands were selected using an IC_50_ threshold of ≤ 1 × 10^3^ nM. For CXCL12, no activity threshold was applied because of the limited number of experimentally validated ligands available in BindingDB (Appendix 2 in the Supplementary File). Consequently, the most potent reported compounds were used for pharmacophore model generation.

The dataset was randomly divided into a training set (80%, n = 40, including references) and an external validation set (20%, n = 10). Chemical redundancy was assessed using canonical SMILES and Tanimoto similarity based on Morgan (ECFP4) fingerprints in ChemToolsHub. No duplicate compounds or closely related analogs (Tanimoto similarity > 0.85) were identified between the training and external validation sets (Appendix 3 in the Supplementary File). Decoys were generated using DecoyFinder 2.0 and the ZINC20 database, with molecular-weight-based selection requiring a minimum standard deviation of 1.00 from the actives and an active-to-decoy ratio of 1:50 (18).

Before pharmacophore model generation, all ligands were energy-minimized using the Merck Molecular Force Field (MMFF94), and conformers were generated using the LigandScout BEST settings, with nitrogen enumeration enabled to account for alternative protonation states of nitrogen-containing heterocycles. A total of 10 models were internally validated using 40 actives and 2000 decoys and externally validated using 10 actives and 500 decoys. Validation parameters included prevalence (PV), true positive rate (TPR), true negative rate (TNR), accuracy (ACC), positive predictive value (PPV), negative predictive value (NPV), false positive rate (FPR), false negative rate (FNR), misclassification rate (MR), goodness of hit score (GH), area under the receiver operating characteristic (ROC) curve (AUC_100_%), and enrichment factor 1% (EF_1_%) (19). The mathematical formulas used to calculate these validation parameters are provided in Appendix 4 in the Supplementary File. The best pharmacophore model was used to screen the 79 test compounds and the reference compounds.

### 3.3. Molecular Docking

Molecular docking studies were performed using AutoDock 4.2.6 and MGLTools 1.5.6. Flexible-rigid docking was used, and the Lamarckian Genetic Algorithm was selected for the conformational search. Before docking, ligand partial charges were assigned by computing Gasteiger charges as implemented in AutoDock 4.2.6 (20).

The docking protocol was validated by re-docking the co-crystallized ligand into the protein. The co-crystallized ligand bound to CXCR4 was (6,6-dimethyl-5,6-dihydroimidazo[2,1-b]thiazol-3-yl)methyl (Z)-N,N'-dicyclohexylcarbamimidothioate (PDB ligand ID: ITD), whereas 1-(4-(1H-tetrazol-5-yl)phenyl)-3-phenylurea (PDB ligand ID: 3GG) was co-crystallized with CXCL12. In this re-docking procedure, the grid box size in the x, y, and z dimensions was set to 40 × 40 × 40 Å grid points, the number of genetic algorithm runs was 100, and the maximum number of energy evaluations was 2.5 × 10^6^ (medium). The x, y, and z coordinates of the grid center for re-docking ITD into CXCR4 were 20.390, -8.747, and 71.653 Å, respectively, whereas those for re-docking 3GG into CXCL12 were 12.296, -7.245, and -1.501 Å, respectively. The validated protocol was then applied to dock the test compounds, and each ligand was docked in triplicate (21). Protein-ligand interactions were visualized using Discovery Studio 2025 (20).

### 3.4. Molecular Dynamics (MD) Simulations

MD simulations were conducted using GROMACS 2021.4 with the AMBER99SB-ILDN force field. Bond lengths were constrained using the LINCS (Linear Constraint Solver) algorithm (22), and ligand topologies were generated using ACPYPE 2023.1.2 with the general AMBER force field 2 (GAFF-2) (23). The system was solvated using the TIP3P water model in a cubic simulation box. To neutralize the system, sodium and chloride ions were added, and energy minimization was performed using the steepest descent algorithm for up to 5 × 10^5^ steps. The system was equilibrated in the NVT ensemble with the V-rescale thermostat at 310 K for 500 ps, followed by equilibration in the NPT ensemble using a Parrinello-Rahman barostat at 1.0 bar and 310 K for 500 ps. Equilibration was assessed by monitoring temperature stability during the NVT phase and pressure stability during the NPT phase, indicating that the target simulation conditions were reached before the production run. Subsequently, production MD was performed for 200 ns with a 2-fs time step, saving 2 × 10^4^ trajectory frames for analysis. Periodic boundary conditions (PBC) were corrected before analysis (22), and each complex was simulated in two replicates. All analyses were conducted using the complete production trajectories without excluding any trajectory segments.

MM-PBSA calculations were performed for the last 10 ns (200 frames sampled every 5 ps). Structural stability and flexibility were evaluated using RMSD, RMSF, Rg, SASA, and H-bond occupancy (24). H-bond occupancies were calculated in VMD 1.9.4 using donor-acceptor distance and angle cutoffs of 3.5 Å and 30°, respectively (25).

### 3.5. Toxicity Prediction

Compound toxicity was predicted using ProTox 3.0. ProTox 3.0 integrates molecular similarity, fragment propensities, the most frequent features, and machine-learning approaches with fragment similarity-based CLUSTER cross-validation. The predicted endpoints included LD_50_, toxicity class, hepatotoxicity, carcinogenicity, immunotoxicity, mutagenicity, and cytotoxicity (26).

### 3.6. Pharmacokinetics and Drug-Likeness Prediction

The pharmacokinetic properties and drug-likeness of the potent 1,2,4-triazole derivatives were evaluated using pkCSM, which applies distance-based graph signatures to predict ADME (absorption, distribution, metabolism, and excretion). Drug-likeness was assessed according to Lipinski's rule of five to evaluate potential oral bioavailability (27).

## 4. Results

### 4.1. Ligand-Based Pharmacophore Modeling

Ligand-based pharmacophore modeling for CXCR4 and CXCL12 generated 10 pharmacophore models for each target. The models were evaluated using internal and external validation, and the validation results were compared to identify the best-performing models. As shown in Appendix 5 in the Supplementary File, model 8 was selected as the optimal pharmacophore model for CXCR4, and model 3 was selected for CXCL12. All selected models achieved an AUC_100_% of ROC ≥ 0.7 (Appendix 6 in the Supplementary File).

Pharmacophore model 8 for CXCR4 comprises four features: two hydrophobic groups, one aromatic ring, and one H-bond donor. By contrast, pharmacophore model 3 for CXCL12 comprises six features: one hydrophobic group, two aromatic rings, and three H-bond acceptors (Appendix 7 in the Supplementary File). These pharmacophore features reflect the dominant interactions required for ligand recognition and are further supported by the superior validation performance of the selected models.

Pharmacophore-based virtual screening indicated that 61 compounds, including mavorixafor, matched the features of model 8, whereas 18 compounds, including LIT-927, matched the features of model 3, as indicated by the pharmacophore-fit scores. Based on these results, 16 derivatives (D41-D51 and D53-D57) that passed virtual screening for both CXCR4 and CXCL12 were advanced to molecular docking (Appendix 8 in the Supplementary File).

### 4.2. Molecular Docking

Validation of the docking method produced RMSD values of 1.58 Å and 0.46 Å for re-docking ITD to CXCR4 and 3GG to CXCL12, respectively (Appendix 9 in the Supplementary File). Molecular docking of the 16 test candidates and reference ligands against CXCR4 and CXCL12 indicated that D54 and D57 had more negative binding free energies than the other candidates and the reference ligands (Table 1), suggesting stronger predicted binding affinities for CXCR4 and CXCL12.

**Table 1.** Calculation of Binding Free Energy from Molecular Docking Results

Compound	Binding Free Energy Against CXCR4 ± SD (kcal/mol)	Binding Free Energy Against CXCL12 ± SD (kcal/mol)
**Mavorixafor**	-9.79 ± 0.13	-
**LIT-927**	-	-4.50 ± 0.00
**D41**	-7.94 ± 0.02	-4.56 ± 0.01
**D42**	-9.54 ± 0.03	-5.22 ± 0.01
**D43**	-9.19 ± 0.04	-6.37 ± 0.06
**D44**	-8.61 ± 0.09	-4.75 ± 0.01
**D45**	-8.54 ± 0.06	-4.60 ± 0.01
**D46**	-8.70 ± 0.04	-5.36 ± 0.00
**D47**	-9.07 ± 0.05	-4.39 ± 0.03
**D48**	-8.50 ± 0.09	-6.11 ± 0.01
**D49**	-9.76 ± 0.00	-5.40 ± 0.01
**D50**	-8.77 ± 0.11	-4.92 ± 0.01
**D51**	-8.82 ± 0.04	-5.86 ± 0.02
**D53**	-9.01 ± 0.02	-4.84 ± 0.01
**D54**	-9.98 ± 0.04	-5.34 ± 0.04
**D55**	-9.13 ± 0.04	-4.78 ± 0.05
**D56**	-9.08 ± 0.12	-5.00 ± 0.01
**D57**	-10.43 ± 0.00	-5.23 ± 0.07

**Figure 2.**
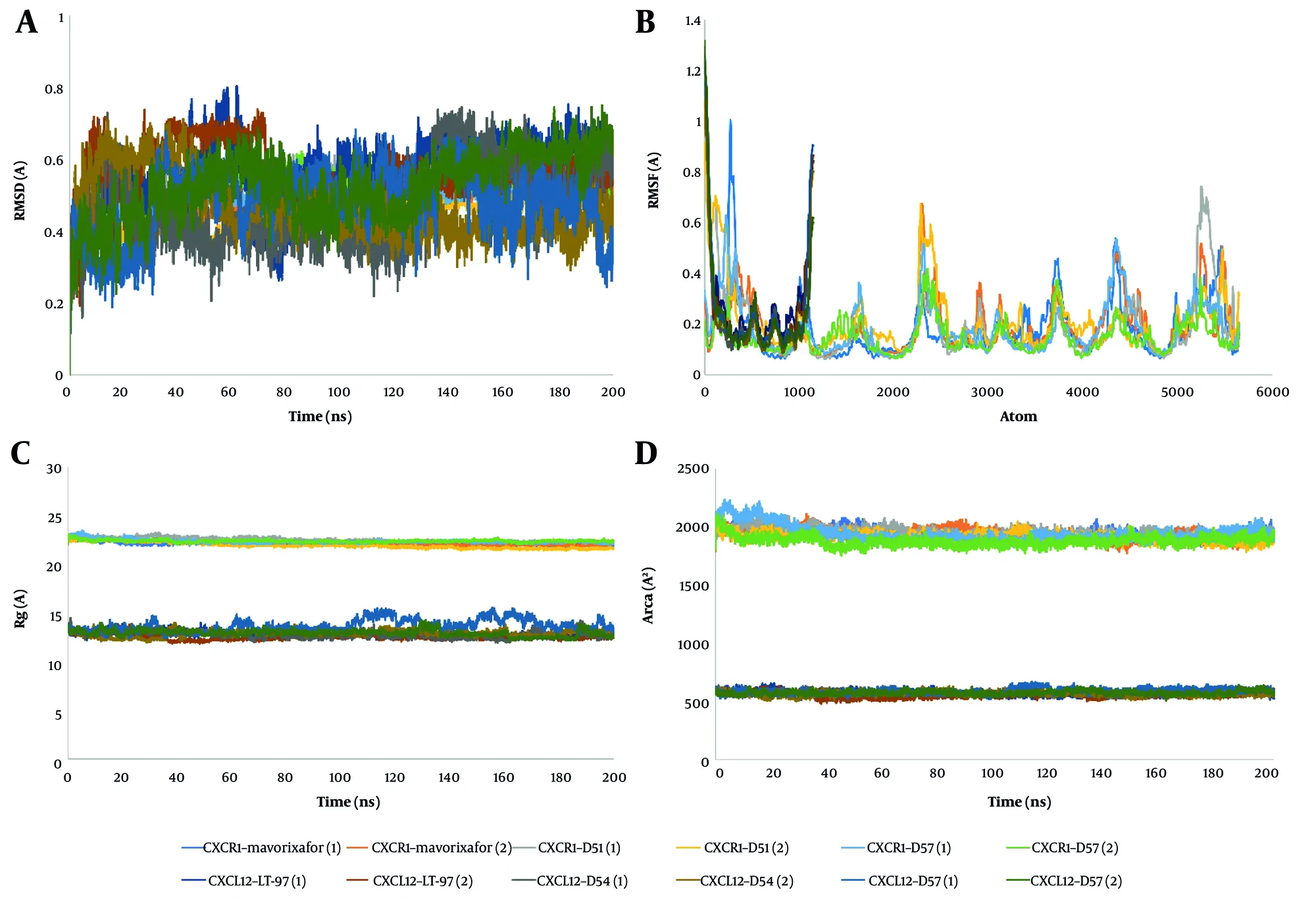
The interactions of protein–ligand in molecular docking study. A, the interactions of CXCR4–mavorixafor, CXCR4 – D54, and CXCR4 – D57; B, the interactions of CXCL12 – LIT-927, CXCL12 – D54, and CXCL12–D57

Across all three docking simulations, the reference ligand mavorixafor interacted with multiple CXCR4 residues, including Glu32, Leu41, Tyr45, Phe93, Trp94, Asp97, Ala98, Trp102, Val112, His113, Tyr116, Ile185, Cys186, Asp187, Ser285, Glu288, and Phe292. Among these, interactions with Tyr45, Trp94, Asp97, Trp102, Val112, His113, Tyr116, and Glu288 were reproducibly observed in all three simulations. Compound D54 consistently interacted with CXCR4 residues Leu41, Tyr45, Trp94, Asp97, Ala98, Trp102, Val112, His113, Tyr116, Arg183, Ile185, Cys186, Asp187, Arg188, Tyr255, Ile284, Ser285, and Glu288. Similarly, D57 showed reproducible interactions with CXCR4 residues Leu41, Tyr45, Trp94, Asp97, Ala98, Trp102, Val112, His113, Tyr116, Arg183, Tyr184, Ile185, Cys186, Asp187, His281, Ser285, and Glu288. Using the same threefold docking procedure, both D54 and D57 also showed affinity for CXCL12, interacting with Glu15, Ser16, His17, Val18, Ala19, Asn22, Leu42, Asn44, Asn45, Arg47, and Val49. The reference ligand LIT-927 also bound to CXCL12 but did not interact with Ser16. In each case, the protein–ligand complexes were stabilized by diverse noncovalent forces, including van der Waals, H-bond, electrostatic, and hydrophobic interactions (Figure 2).

### 4.3. MD Simulations

MD simulations were performed for the CXCR4-mavorixafor, CXCR4-D54, CXCR4-D57, CXCL12-LIT-927, CXCL12-D54, and CXCL12-D57 complexes. Replicate-specific MM-PBSA binding free energies and the corresponding mean RMSD, RMSF, Rg, and SASA values are summarized in Appendix 10 in the Supplementary File, whereas replicate-specific H-bond occupancy data for interactions with occupancies ≥ 0.5% are presented in Appendix 11 in the Supplementary File. Based on two independent MD trajectories, the mean MM-PBSA binding free energies for the CXCR4 complexes were -34.04 ± 4.93, -47.63 ± 0.06, and -32.39 ± 3.88 kcal/mol for D54, D57, and mavorixafor, respectively. The corresponding values for the CXCL12 complexes were -24.21 ± 4.56 kcal/mol for D54, -22.07 ± 1.53 kcal/mol for D57, and -16.86 ± 4.15 kcal/mol for LIT-927. Overall, D54 and D57 exhibited more favorable predicted binding free energies than the reference ligands.

**Figure 3.**
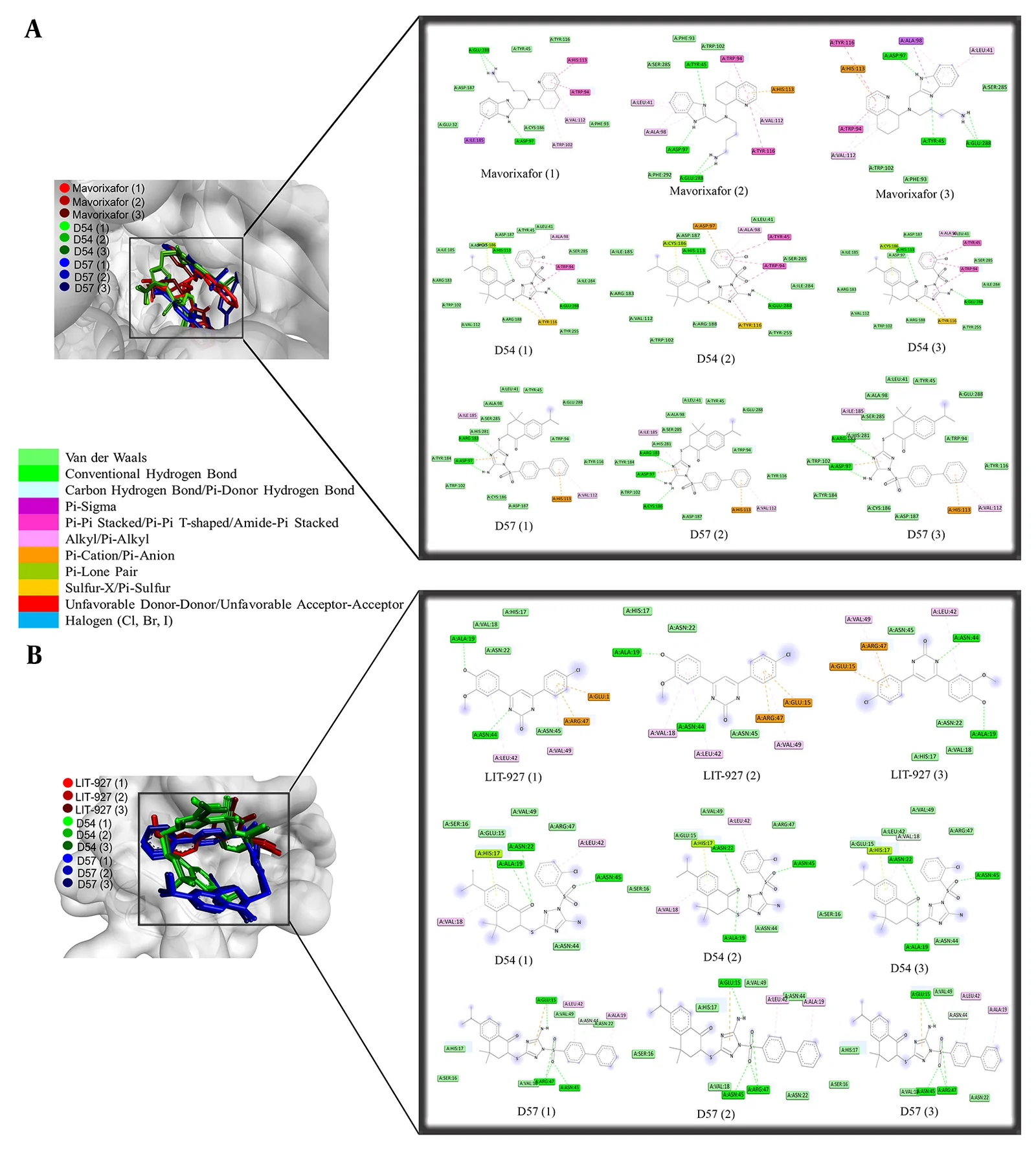
Structural dynamics of CXCR4 and CXCL12 during a 200 ns molecular dynamics simulation. A, root-mean-square deviation (RMSD); B, root-mean-square fluctuations (RMSF); C, radius of gyration (Rg); D, solvent-accessible surface area (SASA)

RMSD analysis showed that all complexes remained below 2 Å throughout the 200-ns simulations, indicating stable protein–ligand complexes without major conformational changes. RMSF analysis indicated that most residues fluctuated by less than 2 Å (Figure 3B). Higher fluctuations were primarily localized to loop regions and terminal ends, which are inherently flexible and less involved in ligand binding (Appendix 10 in the Supplementary File).

The mean Rg and SASA values were also comparable between the two replicates for all complexes (Appendix 10 in the Supplementary File), indicating consistent structural compactness and solvent accessibility throughout the simulations. No increasing trends in Rg or substantial changes in SASA were observed, supporting the overall structural stability of the protein–ligand complexes during the 200-ns simulations (Figure 3C and D).

H-bond occupancy analysis further indicated reproducible protein–ligand interactions across the two independent trajectories (Appendix 11 in the Supplementary File). In the CXCR4 system, mavorixafor consistently formed an H-bond with Glu288, whereas D54 showed the highest occupancy with Asp97 in both replicates. D57 exhibited its most persistent interactions with Asp97 in the first replicate and Arg183 in the second. In the CXCL12 system, LIT-927 interacted predominantly with Glu15 in both replicates, whereas the highest H-bond occupancies for D54 and D57 occurred at different residues, including Lys56, Ala35, Glu63, and Glu15, depending on the replicate.

### 4.4. Toxicity, Pharmacokinetics, and Drug-Likeness Prediction

The results in Table 2 provide a comprehensive evaluation of the toxicity, pharmacokinetic behavior, and drug-likeness properties of D54 and D57.

**Table 2.** Toxicity, Pharmacokinetics, and Drug-Likeness Prediction

Parameter	D54	D57	Requirement
**Toxicity**			
LD_50_	1.5 × 10^3^ mg/kg	1.5 × 10^3^ mg/kg	> 5 × 10^3^ mg/kg
Toxicity Class	4	4	6
Hepatotoxicity	Inactive	Inactive	Inactive
Carcinogenicity	Inactive	Active	Inactive
Immunotoxicity	Active	Inactive	Inactive
Mutagenicity	Inactive	Inactive	Inactive
Cytotoxicity	Inactive	Inactive	Inactive
**Absorption**			
Water solubility	-4.994	-4.41	-
Caco2 permeability	0.532	0.351	> 0.9
Intestinal absorption (human)	89.472	96.112	> 30%
Skin Permeability	-2.736	-2.735	≥ -2.5
P-glycoprotein substrate	No	Yes	-
P-glycoprotein I inhibitor	Yes	Yes	-
P-glycoprotein II inhibitor	Yes	Yes	-
**Distribution**			
VDss (human)	-0.271	-1.148	< -0.15 / > 0.45
Fraction unbound (human)	0	0.16	-
BBB permeability	-0.992	-0.577	< -1 / > 0.3
CNS permeability	-2.11	-1.957	< -3 / > -0.2
**Metabolism**			
CYP2D6 substrate	No	No	-
CYP3A4 substrate	Yes	Yes	-
CYP1A2 inhibitor	No	No	-
CYP2C19 inhibitor	Yes	Yes	-
CYP2C9 inhibitor	Yes	Yes	-
CYP2D6 inhibitor	No	No	-
CYP3A4 inhibitor	Yes	Yes	-
**Excretion**			
Total Clearance (E1)	-0.124	-0.169	Higher is better
Renal OCT2 substrate	No	No	-
**Lipinski's rule of five**			
Mr	505.065	546.718	≤ 500 Da
log P	4.899	5.9126	≤ 5
H-bond acceptors	8	8	≤ 10
H-bond donors	1	1	≤ 5

## 5. Discussion

Initially, 10 pharmacophore models were generated for each target, CXCR4 and CXCL12. During model construction, LigandScout ensured that discarded molecules could not geometrically match the query. Initial alignments were performed using a sophisticated pattern-matching method that allowed flexible inclusion of multiple pharmacophoric features. A database molecule was considered a hit only when all mapped features fell within the tolerance spheres of the corresponding query features. This study used the merged-feature pharmacophore alignment approach, with scoring based on pharmacophore fit and atomic overlap. To assess the structural contributions of active compounds, decoys, and test molecules, all structures were geometrically optimized using MMFF94 until the minimum potential energy was reached, yielding the most stable theoretical conformations (28).

The 10 generated pharmacophore models were validated to select the optimal models. Internal validation assessed model consistency, whereas external validation evaluated generalizability to compounds beyond the training set (16). The requirements for each validation parameter included TPR > 0.5, TNR > 0.5, ACC > 0.5, PPV > 0.5, NPV > 0.5, FPR < 0.5, FNR > 0.5, MR > 0.5, GH 0.7 - 0.8, AUC_100_% ≥ 0.7, and EF_1_%, with higher values considered better (19).

Model 8 was identified as the most robust pharmacophore model for CXCR4, whereas model 3 showed the highest performance for CXCL12. Pharmacophore models can effectively filter large compound libraries to identify hits with features predictive of target activity. Because pharmacophores represent chemical activities and spatial interactions rather than chemical groups, hits are often structurally distinct, which is advantageous for scaffold hopping (28). Compounds D41-D51 and D53-D57 met the screening criteria, with higher pharmacophore-fit scores indicating better alignment.

The RMSD values obtained from docking protocol validation were below 2 Å, indicating excellent agreement with the crystallographic positions of the ligands and supporting the suitability of the method for ligand screening (29). Docking against CXCR4 and CXCL12 showed that D54 and D57 had more negative predicted docking scores than the other compounds and the reference ligands, suggesting more favorable predicted binding (20). The CXCR4-D54 and CXCR4-D57 complexes interacted with all key CXCR4 residues reported as targets for anti-metastatic drugs: Trp94, Asp97, Trp102, Val112, Tyr116, Arg183, Ile185, Cys186, Asp187, and Glu288 (14). The test ligands formed more extensive predicted interactions with CXCR4 than mavorixafor, which did not interact with Arg183. Meanwhile, D54, D57, and LIT-927 interacted with all key CXCL12 residues, including Glu15, Val18, Ala19, Asn22, Leu42, Asn45, Arg47, and Val49 (15). These protein-ligand interactions suggest that D54 and D57 have more favorable predicted docking scores and binding interactions than the reference compounds.

MD simulations were used to investigate the time-dependent behavior of biomolecular systems, in which proteins, ligands, and solvents have full conformational flexibility (30). MM-PBSA analysis showed that D54 and D57 had more negative predicted binding free energies than the reference ligands for both CXCR4 and CXCL12.

The backbone RMSD values consistently converged below 2 Å during MD simulations of the CXCR4-mavorixafor, CXCR4-D54, CXCR4-D57, CXCL12-LIT-927, CXCL12-D54, and CXCL12-D57 complexes. These results indicate an absence of substantial backbone deviations during the MD simulations and demonstrate the overall structural stability of the protein-ligand complexes under the simulated conditions. Among the residues in the active sites of the CXCR4 complexes, Trp94, Asp97, Trp102, Val112, Tyr116, Arg183, Ile185, Cys186, Asp187, and Glu288 showed low RMSF values (< 2 Å), indicating low flexibility in the binding pocket (31). The CXCL12 complexes showed a comparable pattern, with critical interacting residues such as Glu15, Val18, Ala19, Asn22, Leu42, Asn45, Arg47, and Val49 showing smaller fluctuations than surrounding residues. These observations suggest that ligand binding can stabilize the local microenvironment and restrict residue movement within the binding pocket. This behavior is consistent with the interactions observed in both the MD and docking studies. However, the absence of apo (ligand-free) protein simulations is a limitation of this study. To partially address this limitation, reference ligand-protein complexes were included as comparative benchmarks for evaluating the stability of the simulated complexes.

Rg was used to assess molecular compactness and flexibility during the MD simulations. Stable Rg values without a significant increase over time were observed, indicating no structural expansion or unfolding and supporting cohesive, conformationally stable complexes (32). The Rg values of D54 and D57 were comparable to those of the reference ligands, mavorixafor and LIT-927, suggesting that neither compound caused substantial conformational changes in CXCR4 or CXCL12. Although D57 showed a slightly higher Rg in one CXCL12 simulation (13.58 ± 0.61 Å), the variation overlapped with that of the reference ligand and did not indicate systematic or sustained expansion of the protein structure.

SASA was calculated to evaluate solvent accessibility and complex stability. In the CXCR4 and CXCL12 systems, D54 and D57 had SASA values similar to those of the reference ligands, suggesting that both compounds maintained complex stability comparable to that of the reference ligands under the simulated conditions. Higher SASA indicates greater solvent exposure, whereas lower values indicate deeper sequestration within the protein (32). The lower SASA values in the CXCL12 complexes reflect the smaller molecular size of CXCL12 compared with CXCR4.

H-bond occupancy analysis further supported the stability of the protein-ligand complexes. Persistent H-bonds contribute to maintaining spatial configuration throughout a simulation (33). D54, D57, and the reference drugs occupied several active-site residues in CXCR4 and CXCL12, although variability was observed between replicates. These residues included Trp94, Asp97, Arg183, Asp187, and Glu288 in CXCR4 and Glu15, Val18, Ala19, Asn22, Asn45, Arg47, and Val49 in CXCL12. These findings indicate stable ligand retention within the active sites and support the prioritization of D54 and D57 as lead scaffolds for further investigation.

The toxicity and pharmacokinetic profiles of D54 and D57 demonstrated both potential and limitations as oral drug candidates. Both compounds were predicted to be free of hepatotoxic, mutagenic, and cytotoxic risks (26). However, D54 was predicted to exhibit immunotoxicity, whereas D57 showed a positive carcinogenicity prediction, indicating potential safety concerns that require further experimental validation. Despite low solubility and Caco2 permeability, both compounds were predicted to have high intestinal absorption, suggesting that absorption may occur through a transport-mediated mechanism. However, D57, as a P-glycoprotein substrate, may have limited bioavailability relative to D54. The low VDss (< -0.15) indicates that D54 and D57 are predicted to remain more in plasma than in tissues. Moderate BBB and CNS permeability suggest that these compounds may enter the brain without excessive accumulation, supporting their potential to reach metastatic targets. However, violations of Lipinski's rule, particularly the high molecular weight and lipophilicity of D57, together with the predicted toxicity liabilities, may limit their suitability as drug candidates and warrant further optimization and experimental validation (27). Furthermore, the findings of this study are based solely on computational approaches and have not been experimentally validated. Further studies should therefore include cytotoxicity testing and evaluation of anti-metastatic activity using Western blot analysis of the CXCR4/CXCL12 signaling pathway and wound-healing assays to validate the predicted biological activity of D54 and D57.

### 5.1. Conclusions

In conclusion, D54 and D57 emerged as promising lead compounds from computational screening targeting the CXCR4/CXCL12 axis in lung cancer. Their structural frameworks and favorable computational binding profiles support their potential as lead compounds. However, the predicted immunotoxicity of D54, the predicted carcinogenicity of D57, and overall pharmacokinetic and drug-likeness limitations indicate that further structural optimization and experimental validation, including cytotoxicity testing, Western blot analysis, and wound-healing assays, are required before their therapeutic potential can be fully established.

ijpr-25-1-171955-s001.pdf

## Data Availability

All datasets generated and analyzed during this study are available from the corresponding author upon reasonable request.
