# Supplementary material for: Dual Targeting of CXCR4 and CXCL12 by 1,2,4-Triazole Derivatives: A Computational Approach Against Lung Cancer Metastasis
Source: Iran J Pharm Res. 2026 Aug 18;25(1):e171955. doi: 10.5812/ijpr-171955 (PMC13589467; doi:10.5812/ijpr-171955)
Supplement: Supplementary file 1 [file ijpr-25-1-171955-s001.pdf]

Appendix 1. SMILES Strings of the 1,2,4-Triazole Derivatives (D1–D79)

Compound	SMILES String	Ref.
D1	C(OC1=CC=C(C=C1)C2=C3C=C4N=CSC4=CC3=NC5=C2C=CC=C5)C6=NN=C([NH]6)C7=CC=CC=C7	(5)
D2	CC1=CC=C(C=C1)C2=NN=C(COC3=CC=C(C=C3)C4=C5C=C6N=CSC6=CC5=NC7=C4C=CC=C7)[NH]2	(5)
D3	COC1=CC=C(C=C1)C2=NN=C(COC3=CC=C(C=C3)C4=C5C=C6N=CSC6=CC5=NC7=C4C=CC=C7)[NH]2	(5)
D4	COC1=CC(=CC(=C1OC)OC)C2=NN=C(COC3=CC=C(C=C3)C4=C5C=C6N=CSC6=CC5=NC7=C4C=CC=C7)[NH]2	(5)
D5	BrC1=CC=C(C=C1)C2=NN=C(COC3=CC=C(C=C3)C4=C5C=C6N=CSC6=CC5=NC7=C4C=CC=C7)[NH]2	(5)
D6	ClC1=CC=C(C=C1)C2=NN=C(COC3=CC=C(C=C3)C4=C5C=C6N=CSC6=CC5=NC7=C4C=CC=C7)[NH]2	(5)
D7	FC1=CC=C(C=C1)C2=NN=C(COC3=CC=C(C=C3)C4=C5C=C6N=CSC6=CC5=NC7=C4C=CC=C7)[NH]2	(5)
D8	FC(F)(F)C1=CC=C(C=C1)C2=NN=C(COC3=CC=C(C=C3)C4=C5C=C6N=CSC6=CC5=NC7=C4C=CC=C7)[NH]2	(5)
D9	O=[N](=O)C1=CC=C(C=C1)C2=NN=C(COC3=CC=C(C=C3)C4=C5C=C6N=CSC6=CC5=NC7=C4C=CC=C7)[NH]2	(5)
D10	N#CC1=CC=C(C=C1)C2=NN=C(COC3=CC=C(C=C3)C4=C5C=C6N=CSC6=CC5=NC7=C4C=CC=C7)[NH]2	(5)
D11	NC1=NN=C([N]1C2=CC=CC=C2)C3=CC=CC=C3	(6)
D12	NC1=NN=C([N]1C2=CC=C(Cl)C=C2)C3=CC=CC=C3	(6)
D13	NC1=NN=C([N]1C2=CC=C(Cl)C=C2)C3=CC=C(Br)C=C3	(6)
D14	NC1=NN=C([N]1C2=CC=C(Cl)C=C2)C3=CC=C(Cl)C=C3	(6)
D15	NC1=NN=C([N]1C2=CC=C(Cl)C=C2)C3=CC=C(F)C=C3	(6)
D16	C[S](=O)(=O)C1=CC=C(C=C1)C2=NN=C(N)[N]2C3=CC=C(Cl)C=C3	(6)
D17	NC1=NN=C([N]1C2=CC=CC=C2)C3=CC=C(Br)C=C3	(6)
D18	NC1=NN=C([N]1C2=CC=CC=C2)C3=CC=C(Cl)C=C3	(6)
D19	NC1=NN=C([N]1C2=CC=CC=C2)C3=CC=C(F)C=C3	(6)
D20	CC1=CC=C(C=C1)C2=NN=C(N)[N]2C3=CC=CC=C3	(6)
D21	NC1=NN=C([N]1C2=CC=CC=C2)C3=CC=C(C=C3)C(F)(F)F	(6)
D22	C[N+]1=C([O-])C2=C(N=C[N]2CC3=NN=C(SCC(=O)NC4=CC=CC=C4)[N]3C5=CC=CC=C5)[N+](=C1[O-])C	(10)
D23	C[N+]1=C([O-])C2=C(N=C[N]2CC3=NN=C(SCC(=O)NC4=C(Cl)C=C(Cl)C=C4)[N]3C5=CC=CC=C5)[N+](=C1[O-])C	(10)
D24	CC1=CC=C(NC(=O)CSC2=NN=C(C[N]3C=NC4=C3C(=[N+](C)C(=[N+]4C)[O-])[O-])[N]2C5=CC=CC=C5)C(=C1)C	(10)
D25	C[N+]1=C([O-])C2=C(N=C[N]2CC3=NN=C(SCC(=O)NC4=CC=C(Cl)C(=C4)Cl)[N]3C5=CC=CC=C5)[N+](=C1[O-])C	(10)
D26	C[N+]1=C([O-])C2=C(N=C[N]2CC3=NN=C(SCC(=O)NC4=CC=C(F)C=C4)[N]3C5=CC=CC=C5)[N+](=C1[O-])C	(10)
D27	C[N+]1=C([O-])C2=C(N=C[N]2CC3=NN=C(SCC(=O)NC4=CC=CC=C4Cl)[N]3C5=CC=CC=C5)[N+](=C1[O-])C	(10)
D28	CC1=CC=C(NC(=O)CSC2=NN=C(C[N]3C=NC4=C3C(=[N+](C)C(=[N+]4C)[O-])[O-])[N]2C5=CC=CC=C5)C=C1	(10)
D29	CC1=CC=C(NC(=O)CSC2=NN=C(C[N]3C=NC4=C3C(=[N+](C)C(=[N+]4C)[O-])[O-])[N]2C5=CC=CC=C5)C=C1C	(10)
D30	C[N+]1=C([O-])C2=C(N=C[N]2CC3=NN=C(SCC(=O)NC4=CC=C(Cl)C=C4)[N]3C5=CC=CC=C5)[N+](=C1[O-])C	(10)
D31	C[N+]1=C([O-])C2=C(N=C[N]2CC3=NN=C(SCC(=O)NC4=CC=CC=C4F)[N]3C5=CC=CC=C5)[N+](=C1[O-])C	(10)
D32	N[N]1C(=NN=C1CSC2=NN=C3CC4=C(S[NH2++]C=C4)[N]23)S	(4)
D33	SC1=NN=C(CSC2=NN=C3CC4=C(S[NH2++]C=C4)[N]23)S1	(4)
D34	N[N]1C(=NN=C1CSC2=NN=C3CC4=C(S[NH2++]C=C4)[N]23)S	(4)
D35	OC1=NN=C(CSC2=NN=C3C=C4CC=NSC4[N]23)O1	(4)
D36	NC1=NN=C(CSC2=NN=C3CC4=C(S[NH2++]C=C4)[N]23)S1	(4)
D37	SC1=NN=C(CSC2=NN=C3CC4=C(S[NH2++]C=C4)[N]23)[NH]1	(4)
D38	CC1=N[N](C(=O)CSC2=NN=C3C=C4CC=NSC4[N]23)C(=C1)C	(4)
D39	CC1=NN(C(=O)CSC2=NN=C3C=C4CC=NSC4[N]23)C(=O)C1	(4)
D40	NC1=C(C=N[N]1C(=O)CSC2=NN=C3C=C4CC=NSC4[N]23)C#N	(4)
D41	CC(C)C1=CC2=C(C=C1)C(=O)C(CC2(C)C)SC3=N[N](C(=N3)N)[S](=O)(=O)C4=C(C)C=C(C)C=C4C	(8)

Compound	SMILES String	Ref.
D42	CC(C)C1=CC2=C(C=C1)C(=O)C(CC2(C)C)SC3=N[N](C(=N3)N)[S](=O)(=O)C4=CC(=CC=C4C)C	(8)
D43	CC(C)C1=CC2=C(C=C1)C(=O)C(CC2(C)C)SC3=N[N](C(=N3)N)[S](=O)(=O)C4=CC=CC=C4[N](=O)=O	(8)
D44	CC(C)C1=CC2=C(C=C1)C(=O)C(CC2(C)C)SC3=N[N](C(=N3)N)[S](=O)(=O)C4=CC=CC(=C4)Br	(8)
D45	CC(C)C1=CC2=C(C=C1)C(=O)C(CC2(C)C)SC3=N[N](C(=N3)N)[S](=O)(=O)C4=CC=C(C=C4)C(F)(F)F	(8)
D46	COC1=CC=C(C=C1)[S](=O)(=O)[N]2N=C(SC3CC(C)(C)C4=C(C=CC(=C4)C(C)C)C3=O)N=C2N	(8)
D47	CC(C)C1=CC2=C(C=C1)C(=O)C(CC2(C)C)SC3=N[N](C(=N3)N)[S](=O)(=O)C4=C(F)C(=C(F)C(=C4F)F) F	(8)
D48	CC(C)C1=CC2=C(C=C1)C(=O)C(CC2(C)C)SC3=N[N](C(=N3)N)[S](=O)(=O)C4=CC=C(Cl)C(=C4)[N](=O)=O	(8)
D49	CC(C)C1=CC2=C(C=C1)C(=O)C(CC2(C)C)SC3=N[N](C(=N3)N)[S](=O)(=O)C4=CC=CC=C4	(8)
D50	CC(C)C1=CC2=C(C=C1)C(=O)C(CC2(C)C)SC3=N[N](C(=N3)N)[S](=O)(=O)C4=CC=C(Cl)C=C4	(8)
D51	CC(C)C1=CC2=C(C=C1)C(=O)C(CC2(C)C)SC3=N[N](C(=N3)N)[S](=O)(=O)C4=CC=CC(=C4)[N](=O)=O	(8)
D52	CC(C)C1=CC2=C(C=C1)C(=O)C(CC2(C)C)SC3=N[N](C(=N3)N)[S](=O)(=O)C4=CC=C(C=C4)[N](=O)=O	(8)
D53	CC(C)C1=CC2=C(C=C1)C(=O)C(CC2(C)C)SC3=N[N](C(=N3)N)[S](=O)(=O)C4=CC=C(C)C=C4	(8)
D54	CC(C)C1=CC2=C(C=C1)C(=O)C(CC2(C)C)SC3=N[N](C(=N3)N)[S](=O)(=O)C4=CC=CC=C4Cl	(8)
D55	CC(C)C1=CC2=C(C=C1)C(=O)C(CC2(C)C)SC3=N[N](C(=N3)N)[S](=O)(=O)C4=CC=C(Br)C=C4	(8)
D56	CC(C)C1=CC2=C(C=C1)C(=O)C(CC2(C)C)SC3=N[N](C(=N3)N)[S](=O)(=O)C4=CC=C(F)C=C4F	(8)
D57	CC(C)C1=CC2=C(C=C1)C(=O)C(CC2(C)C)SC3=N[N](C(=N3)N)[S](=O)(=O)C4=CC=C(C=C4)C5=CC=C C=C5	(8)
D58	COC1=C(OC)C(=CC(=C1)C2=NSC(=N2)C3=CC=C(C=C3)C4=N[N](C(=O)C5=CC=CC=C5)C(=N4)C6=C C(=C(OC)C(=C6)OC)OC)OC	(9)
D59	COC1=C(OC)C(=CC(=C1)C(=O)[N]2N=C(N=C2C3=CC(=C(OC)C(=C3)OC)OC)C4=CC=C(C=C4)C5=NC(=NS5)C6=CC(=C(OC)C(=C6)OC)OC)OC	(9)
D60	COC1=CC(=CC(=C1)C(=O)[N]2N=C(N=C2C3=CC(=C(OC)C(=C3)OC)OC)C4=CC=C(C=C4)C5=NC(=NS 5)C6=CC(=C(OC)C(=C6)OC)OC)OC	(9)
D61	COC1=CC=C(C=C1)C(=O)[N]2N=C(N=C2C3=CC(=C(OC)C(=C3)OC)OC)C4=CC=C(C=C4)C5=NC(=NS5)C6=CC(=C(OC)C(=C6)OC)OC	(9)
D62	COC1=C(OC)C(=CC(=C1)C2=NSC(=N2)C3=CC=C(C=C3)C4=N[N](C(=O)C5=CC=C(C=C5)[N](=O)=O)C (=N4)C6=CC(=C(OC)C(=C6)OC)OC)OC	(9)
D63	COC1=C(OC)C(=CC(=C1)C2=NSC(=N2)C3=CC=C(C=C3)C4=N[N](C(=O)C5=CC(=CC(=C5)[N](=O)=O)[N](=O)=O)C(=N4)C6=CC(=C(OC)C(=C6)OC)OC)OC	(9)
D64	COC1=C(OC)C(=CC(=C1)C2=NSC(=N2)C3=CC=C(C=C3)C4=N[N](C(=O)C5=CC=C(Cl)C=C5)C(=N4)C6 =CC(=C(OC)C(=C6)OC)OC)OC	(9)
D65	COC1=C(OC)C(=CC(=C1)C2=NSC(=N2)C3=CC=C(C=C3)C4=N[N](C(=O)C5=CC=C(Br)C=C5)C(=N4)C6 =CC(=C(OC)C(=C6)OC)OC)OC	(9)
D66	COC1=C(OC)C(=CC(=C1)C2=NSC(=N2)C3=CC=C(C=C3)C4=N[N](C(=O)C5=CC=C(C=C5)C#N)C(=N4) C6=CC(=C(OC)C(=C6)OC)OC)OC	(9)
D67	COC1=C(OC)C(=CC(=C1)C2=NSC(=N2)C3=CC=C(C=C3)C4=N[N](C(=O)C5=CC=C(C)C=C5)C(=N4)C6 =CC(=C(OC)C(=C6)OC)OC)OC	(9)
D68	COC1=CC=C(C=C1)[N]2C(=NN=C2C3=CC=CC=C3)SCC(=O)C4=CC=CC=C4	(7)
D69	COC1=CC=C(C=C1)[N]2C(=NN=C2C3=CC=CC=C3)SCC(=O)C4=CC(=C(OC)C(=C4)OC)OC	(7)
D70	COC1=CC=C(C=C1)[N]2C(=NN=C2C3=CC=CC=C3)SCC(=O)C4=CC=C(Cl)C=C4	(7)
D71	COC1=CC=C(C=C1)[N]2C(=NN=C2C3=CC(=C(OC)C(=C3)OC)OC)SCC(=O)C4=CC=CC=C4	(7)
D72	COC1=CC=C(C=C1)[N]2C(=NN=C2C3=CC(=C(OC)C(=C3)OC)OC)SCC(=O)C4=CC(=C(OC)C(=C4)OC) OC	(7)
D73	COC1=CC=C(C=C1)[N]2C(=NN=C2C3=CC(=C(OC)C(=C3)OC)OC)SCC(=O)C4=CC=C(Cl)C=C4	(7)
D74	COC1=CC=C(C=C1)[N]2C(=NN=C2C3=CC(=C(OC)C(=C3)OC)OC)S	(7)
D75	NC(=S)NNC(=O)CSC1=NN=C2CC3=C(S[NH2++]C=C3)[N]12	(11)
D76	O=C1CC(=O)N(N1)C(=O)CSC2=NN=C3CC4=C(S[NH2++]C=C4)[N]23	(11)
D77	OC1=CC=C(O)[N]1NC(=O)CSC2=NN=C3C=C4CC=NSC4[N]23	(11)
D78	OC1=C2C=CC=CC2=C(O)[N]1NC(=O)CSC3=NN=C4C=C5CC=NSC5[N]34	(11)
D79	OC1=C(NNC(=O)CSC2=NN=C3C=C4CC=NSC4[N]23)C5=C([NH]1)C=CC=C5	(11)

Appendix 2. Biological Activity of the Active Training and External Validation Sets

No.	SMILES String	Biological Activity
**Active Training Set for CXCR4**		
1	[nH]1c(nc2c1cccc2)CN([C@@H]4c3ncccc3CCC4)CCCCN (mavorixafor)	IC_{50}: 13
2	[O-][n+]1cccnc1NCc1ccc(CNc2nccc[n+]2[O-])cc1	IC_{50}: 1×10^3

3	C(Nc1ccccc1)[C@H]1CC[C@@H](CNc2ccccc2)CC1	IC_{50}: 1×10^3
4	C(SC(NC1CCCCC1)=N/C1CCCCC1)C1=CSC2=NCCN12	IC_{50}: 11
5	c1ccc(cc1)-c1nc([nH]c1-c1ccccc1)-c1ccc(cc1)-c1nc(c([nH]1)-c1ccccc1)-c1ccccc1	IC_{50}: 1×10^3
6	CC4(C)Cn3c(CS/C(=N/C1CCCCC1)NC2CCCCC2)csc3=N4	IC_{50}: 10
7	Clc1nc(Cl)nc(n1)NCc2ccc(cc2)CNc3nc(Cl)nc(Cl)n3	IC_{50}: 10
8	FC(F)(F)c3cc(NCc1ccc(cc1)CNc2cc(ccc2)C(F)(F)F)ccc3	IC_{50}: 1×10^3
9	Fc1nc(F)nc(n1)NCc2ccc(cc2)CNc3nc(F)nc(F)n3	IC_{50}: 100
10	Fc2cnc(NCc1ccc(cc1)CO)cc2	IC_{50}: 1×10^3
11	Fc3c(NCc1ccc(cc1)CNc2ncccn2)cccc3	IC_{50}: 100
12	Fc3cc(NCc1ccc(cc1)CNc2cc(F)ccc2)ccc3	IC_{50}: 100
13	Fc3ccc(NCc1ccc(cc1)CNc2ccc(F)cc2)cc3	IC_{50}: 1×10^3
14	Fc3cnc(NCc1ccc(cc1)CNc2ncc(cc2)C)cc3	IC_{50}: 100
15	N(c1ccccc1)(Cc2ccc(cc2)CN(c3ccccc3)C)C	IC_{50}: 100
16	N(c1ccccc1)Cc2c(c(c(c(c2C)C)CNc3ccccc3)C)C	IC_{50}: 10
17	N(c1ccccc1)Cc2c4c(c(cc2)CNc3ccccc3)cccc4	IC_{50}: 100
18	N(c1ccccc1)Cc2c5c(c(c3c2cccc3)CNc4ccccc4)cccc5	IC_{50}: 100
19	N(c1ccccc1)Cc2cc(cc(c2)CNc3ccccc3)CNc4ccccc4	IC_{50}: 1×10^3
20	N(c1ccccc1)CCc2ccc(cc2)CCNc3ccccc3	IC_{50}: 100
21	N(c1ccccc1)CCNCc2ccc(cc2)CNCCNc3ccccc3	IC_{50}: 1×10^3
22	N(Cc1ccc(cc1)CN(C)C)(C)C	IC_{50}: 1×10^3
23	N(CCNCc1ccc(cc1)CNCCN(C)C)(C)C	IC_{50}: 1×10^3
24	N=C(N)NCCC[C@@H]1NC(=O)[C@H](CCCNC(=N)N)NC(=O)[C@@H](Cc2ccc(O)cc2)NC(=O)CNC(=O)[C@H](c2ccc3ccccc3c2)NC1=O	IC_{50}: 109
25	n1c(cccc1)CNCc2ccc(cc2)CNCc3ncccc3	IC_{50}: 1×10^3
26	n1c(nccc1)NCc2ccc(cc2)CNc3ncccc3	IC_{50}: 100
27	n1cc(ccc1)CNCc2ccc(cc2)CNCc3cnccc3	IC_{50}: 1×10^3
28	n1ccc(cc1)CNCc2ccc(cc2)CNCc3ccncc3	IC_{50}: 1×10^3
29	N3(Cc1ccc(cc1)CN2CCNCCCNCCNCCC2)CCNCCCNCCNCCC3	IC_{50}: 10
30	N3=C(NCc1ccc(cc1)CNC2=NCCN2)NCC3	IC_{50}: 1×10^3
31	O(c1ccccc1)Cc2ccc(cc2)COc3ccccc3	IC_{50}: 1×10^3
32	O(c1ncccc1)Cc2ccc(cc2)CNc3ncccn3	IC_{50}: 100
33	O(c1ncccc1)Cc2ccc(cc2)COc3ncccc3	IC_{50}: 1×10^3
34	O(c2cc(NCc1ccc(cc1)CO)ccc2)C	IC_{50}: 1×10^3
35	O(c3c(NCc1ccc(cc1)CNc2c(OC)cccc2)cccc3)C	IC_{50}: 10
36	O(c3cc(NC(=O)c1ccc(cc1)C(=O)Nc2cc(OC)ccc2)ccc3)C	IC_{50}: 1×10^3
37	O(Cc1ccc(cc1)CNc2ncccn2)C	IC_{50}: 1×10^3
38	O=C(Nc1ccccc1)c2cc(cc(c2)CNc3ccccc3)CNc4ccccc4	IC_{50}: 100
39	O=C(O)c3cc(NCc1ccc(cc1)CNc2ccccc2)ccc3	IC_{50}: 100
40	S(c1ccccc1)Cc2ccc(cc2)CSc3ccccc3	IC_{50}: 1×10^3
**External Validation Set for CXCR4**		
1	Cc1cc(CN)c(C)cc1CN	IC_{50}: 1×10^3
2	Oc1c(cc(O)c(c1)CN2CCOCC2)CN3CCOCC3	IC_{50}: 1×10^3
3	N(c1ccccc1)Cc2c(cc(c(c2)C)CNc3ccccc3)C	IC_{50}: 100
4	Fc3c(NCc1ccc(cc1)CNc2c(F)cccc2)cccc3	IC_{50}: 10
5	N(Cc1ccc(cc1)CNCc2ccccc2)Cc3ccccc3	IC_{50}: 100
6	Oc3ccc(NCc1ccc(cc1)CNc2ccc(O)cc2)cc3	IC_{50}: 1×10^3
7	N(c1ccccc1)Cc2c(cccc2)CNc3ccccc3	IC_{50}: 100
8	[N+](c3ccc(NCc1ccc(cc1)CNc2ccc(cc2)C#N)cc3)#[C-]	IC_{50}: 1×10^3
9	N(c1ccccc1)[C@H](c2ccc(cc2)[C@H](Nc3ccccc3)C)C	IC_{50}: 100
10	O=[N+]([O-])c3ccc(NCc1ccc(cc1)CNc2ccc([N+](=O)[O-])cc2)cc3	IC_{50}: 1×10^3
**Active Training Set for CXCL12**		
1	Clc1ccc(cc1)C(=O)C=Cc1ccc2OCCOc2c1	Ki: 1.38×10^3
2	Clc3ccc(c2[nH]c(=O)nc(c1cc(OC)c(O)cc1)c2)cc3 (LIT-927)	Ki: 267
3	CNc1nc(cc(n1)-c1ccc(O)c(OC)c1)-c1ccc(Cl)cc1	Ki: $>1 \times 10^4$
4	COc1cc(C=C(/C)C(=O)c2ccc(Cl)cc2)ccc1O	Ki: $>5 \times 10^3$
5	COc1cc(C=C2/Cc3cc(Cl)ccc3C2=O)ccc1O	Ki: 107
6	COc1cc(C=C2/COc3cc(Cl)ccc3C2=O)ccc1O	Ki: 357
7	COc1cc(C=C2/Oc3cc(Cl)ccc3C2=O)ccc1O	Ki: 203
8	COc1cc(C=CC(=NO)c2ccc(Cl)cc2)ccc1O	Ki: $>1 \times 10^4$
9	COc1cc(C=CC(=O)c2c(Cl)cccc2Cl)ccc1O	Ki: 71
10	COc1cc(C=CC(=O)c2cc(Cl)ccc2Cl)ccc1O	Ki: 53
11	COc1cc(C=CC(=O)c2ccc(cc2)C(C)C)ccc1O	Ki: 25

12	COc1cc(C=CC(=O)c2ccc(cc2)C(O)=O)ccc1O	Ki: 5.35×10^3
13	COc1cc(C=CC(=O)c2ccc(Cl)c(Cl)c2)ccc1O	Ki: 714
14	COc1cc(C=CC(=O)c2ccc(Cl)cc2)ccc1[N+]([O-])=O	Ki: $>1 \times 10^4$
15	COc1cc(C=CC(=O)c2ccc(Cl)cc2Cl)ccc1O	Ki: 48
16	COc1cc(C=CC(=O)c2ccc(Cl)cc2F)ccc1O	Ki: 107
17	COc1cc(C=CC(=O)c2cccc(Cl)c2Cl)ccc1O	Ki: 357
18	COc1cc(C=CC(=O)c2ccccc2)ccc1O	Ki: $>1 \times 10^4$
19	COc1cc(C=CC(=O)c2ccccc2Cl)ccc1O	Ki: 107
20	COc1cc(C=CC(=O)c2cccnc2)ccc1O	Ki: $>1 \times 10^4$
21	COc1cc(C=CC(=O)c2ccncc2)ccc1O	Ki: $>1 \times 10^4$
22	COc1cc(ccc1O)C(=O)C=Cc1ccc(Cl)cc1	Ki: $>1 \times 10^4$
23	COc1cc(ccc1O)C1CC(=NN1)c1ccc(Cl)cc1	Ki: 107
24	COc1cc(ccc1O)C1CC(=NN1C(C)=O)c1ccc(Cl)cc1	Ki: $>1 \times 10^4$
25	COc1cc(ccc1O)-c1cc(n[nH]1)-c1ccc(Cl)cc1	Ki: $>1 \times 10^4$
26	COc1cc(ccc1O)-c1cc(nc(=O)[nH]1)-c1ccc(Cl)cc1	Ki: 267
27	COc1cc(ccc1O)-c1cc(nc(N)n1)-c1ccc(Cl)cc1	Ki: $>1 \times 10^4$
28	COc1ccc(C=CC(=O)c2ccc(Cl)cc2)cc1OC	Ki: $>1 \times 10^4$
29	COc1ccc(cc1)C(=O)C=Cc1ccc(O)c(OC)c1	Ki: 1.78×10^3
30	COc1cccc(C=CC(=O)c2ccc(Cl)cc2)c1	Ki: $>1 \times 10^4$
31	COc3cc(c2cc(c1ccc(Cl)cc1)nc(C)n2)ccc3O	Ki: 1.68×10^3
32	NCCOCCOc1cc(C=C2/Cc3cc(Cl)ccc3C2=O)ccc1O	Ki: 496
33	NCCOCCOc1cc(C=CC(=O)c2ccc(Cl)cc2)ccc1O	Ki: 1.07×10^3
34	NCCOCCOCCOc1cc(C=CC(=O)c2ccc(Cl)cc2)ccc1O	Ki: 475
35	O=C(c1ccccc1)\C=C\c2ccccc2	IC_{50}: 5×10^5
36	O=C(CCc1ccccc1)c1ccccc1	IC_{50}: 1.8×10^5
37	OC(=O)c1cc(C=CC(=O)c2ccc(Cl)cc2)ccc1O	Ki: $>1 \times 10^4$
38	Oc1ccc(C=CC(=O)c2ccc(Cl)cc2)cc1	Ki: 625
39	Oc1ccc(C=CC(=O)c2ccc(Cl)cc2)cc1O	Ki: 350
40	Oc1ccc(C=CC(=O)c2ccccc2O)cc1	IC_{50}: 2.42×10^4
**External Validation Set for CXCL12**		
1	COc1cc(C=CC(=O)c2ccc(C)cc2)ccc1O	Ki: 285
2	COc1cc(C=CC(=O)c2ccc(Br)cc2)ccc1O	Ki: 44
3	COc1cc(C=CC(=O)c2ccc(I)cc2)ccc1O	Ki: 107
4	COc1ccc(C=CC(=O)c2ccc(Cl)cc2)cc1O	Ki: $>1 \times 10^4$
5	COc1cc(C=CC(=O)c2ccc(Cl)cc2)ccc1N	Ki: $>1 \times 10^4$
6	COc1cc(CCC(=O)c2ccc(Cl)cc2)ccc1O	Ki: $>1 \times 10^4$
7	COc1cc(C=CC(=O)c2ccc(Cl)cc2)ccc1O	Ki: 53
8	COc1cc(C=CC(=O)c2ccc(F)cc2)ccc1O	Ki: 430
9	COc1cc(ccc1O)C(C)=CC(=O)c1ccc(Cl)cc1	Ki: $>1 \times 10^4$
10	COc1cc(C=CC(=O)c2ccc(cc2)C(F)(F)F)ccc1O	Ki: 1070

Appendix 3. Assessment of Chemical Redundancy Between the Training and External Validation Sets Using Canonical SMILES and Morgan (ECFP4) Tanimoto Similarity

Tanimoto Similarity of Active Compounds Targeting CXCR4

**Training Set** \ **External Validation Set**	**1**	**2**	**3**	**4**	**5**	**6**	**7**	**8**	**9**	**10**
**1**	0.078	0.054	0.085	0.101	0.094	0.057	0.091	0.049	0.072	0.053
**2**	0.075	0.061	0.186	0.316	0.294	0.297	0.211	0.229	0.116	0.293
**3**	0.091	0.098	0.375	0.229	0.276	0.242	0.444	0.182	0.281	0.211
**4**	0.068	0.108	0.076	0.061	0.067	0.079	0.065	0.053	0.046	0.058
**5**	0.054	0.043	0.146	0.122	0.176	0.073	0.167	0.058	0.158	0.065
**6**	0.082	0.087	0.088	0.058	0.063	0.076	0.062	0.051	0.060	0.070
**7**	0.125	0.071	0.189	0.344	0.321	0.367	0.219	0.268	0.108	0.314
**8**	0.105	0.085	0.316	0.361	0.303	0.382	0.324	0.289	0.175	0.333
**9**	0.091	0.098	0.189	0.433	0.321	0.367	0.219	0.268	0.108	0.314
**10**	0.071	0.102	0.152	0.333	0.243	0.316	0.171	0.245	0.087	0.250

**11**	0.073	0.082	0.209	0.759*	0.324	0.289	0.237	0.224	0.140	0.256
**12**	0.114	0.116	0.343	0.438	0.333	0.419	0.355	0.310	0.189	0.361
**13**	0.094	0.100	0.303	0.448	0.333	0.600	0.357	0.417	0.143	0.500
**14**	0.122	0.078	0.200	0.325	0.237	0.275	0.167	0.240	0.109	0.244
**15**	0.129	0.073	0.229	0.235	0.333	0.176	0.226	0.133	0.212	0.154
**16**	0.161	0.071	0.571	0.265	0.321	0.323	0.560	0.238	0.323	0.278
**17**	0.125	0.098	0.571	0.265	0.321	0.323	0.696	0.238	0.281	0.278
**18**	0.094	0.073	0.483	0.273	0.333	0.333	0.583	0.244	0.290	0.286
**19**	0.133	0.103	0.556	0.367	0.400	0.444	0.609	0.316	0.300	0.375
**20**	0.094	0.073	0.387	0.355	0.385	0.333	0.462	0.244	0.290	0.286
**21**	0.086	0.068	0.394	0.406	0.560	0.387	0.464	0.286	0.265	0.333
**22**	0.160	0.086	0.156	0.200	0.250	0.214	0.143	0.154	0.133	0.182
**23**	0.125	0.071	0.189	0.265	0.423	0.281	0.182	0.209	0.139	0.243
**24**	0.069	0.089	0.104	0.120	0.130	0.171	0.096	0.081	0.079	0.101
**25**	0.083	0.067	0.205	0.314	0.538	0.257	0.235	0.196	0.158	0.225
**26**	0.079	0.064	0.225	0.371	0.355	0.314	0.257	0.239	0.150	0.275
**27**	0.086	0.068	0.179	0.286	0.560	0.265	0.206	0.200	0.132	0.231
**28**	0.100	0.077	0.171	0.290	0.545	0.310	0.200	0.225	0.118	0.265
**29**	0.079	0.220	0.089	0.143	0.167	0.150	0.100	0.140	0.070	0.133
**30**	0.081	0.089	0.171	0.306	0.281	0.324	0.194	0.273	0.098	0.282
**31**	0.100	0.077	0.206	0.250	0.360	0.226	0.241	0.167	0.188	0.162
**32**	0.071	0.059	0.205	0.333	0.314	0.316	0.231	0.220	0.136	0.250
**33**	0.088	0.070	0.154	0.222	0.267	0.200	0.176	0.128	0.135	0.146
**34**	0.119	0.098	0.310	0.286	0.263	0.368	0.282	0.260	0.182	0.295
**35**	0.111	0.065	0.263	0.516	0.367	0.324	0.265	0.244	0.184	0.282
**36**	0.100	0.082	0.209	0.109	0.125	0.140	0.175	0.091	0.195	0.125
**37**	0.105	0.063	0.220	0.324	0.303	0.343	0.216	0.234	0.146	0.268
**38**	0.098	0.102	0.395	0.268	0.278	0.351	0.412	0.245	0.220	0.310
**39**	0.098	0.102	0.395	0.333	0.353	0.429	0.412	0.298	0.220	0.375
**40**	0.100	0.077	0.206	0.250	0.360	0.188	0.241	0.140	0.188	0.162

Tanimoto Similarity of Active Compounds Targeting CXCL12

External Validation Set / Training Set	1	2	3	4	5	6	7	8	9	10
**1**	0.385	0.377	0.377	0.521	0.510	0.237	0.521	0.377	0.309	0.357
**2**	0.283	0.279	0.279	0.322	0.274	0.393	0.393	0.279	0.400	0.266
**3**	0.276	0.271	0.271	0.316	0.267	0.389	0.389	0.271	0.396	0.279
**4**	0.480	0.500	0.471	0.500	0.462	0.500	0.630	0.471	0.574	0.444
**5**	0.362	0.356	0.356	0.356	0.328	0.379	0.455	0.356	0.386	0.339
**6**	0.344	0.339	0.339	0.339	0.313	0.339	0.431	0.339	0.367	0.323
**7**	0.362	0.356	0.356	0.356	0.328	0.356	0.455	0.356	0.386	0.339
**8**	0.531	0.520	0.520	0.551	0.510	0.434	0.689	0.520	0.500	0.491
**9**	0.609	0.596	0.596	0.531	0.490	0.364	0.667	0.596	0.423	0.560
**10**	0.583	0.571	0.571	0.571	0.529	0.400	0.711	0.571	0.462	0.538
**11**	0.744	0.727	0.727	0.617	0.571	0.382	0.767	0.727	0.471	0.681
**12**	0.800	0.780	0.780	0.622	0.574	0.377	0.780	0.780	0.469	0.727
**13**	0.705	0.689	0.689	0.617	0.571	0.407	0.767	0.689	0.471	0.646
**14**	0.529	0.519	0.519	0.646	0.667	0.339	0.681	0.519	0.418	0.491
**15**	0.583	0.571	0.571	0.571	0.529	0.400	0.711	0.571	0.462	0.538
**16**	0.560	0.549	0.549	0.549	0.509	0.386	0.681	0.612	0.444	0.547
**17**	0.571	0.560	0.560	0.500	0.463	0.345	0.625	0.560	0.400	0.528
**18**	0.775	0.756	0.756	0.600	0.553	0.358	0.756	0.756	0.449	0.705
**19**	0.596	0.583	0.583	0.520	0.481	0.357	0.652	0.583	0.415	0.549
**20**	0.638	0.625	0.625	0.500	0.463	0.322	0.625	0.625	0.375	0.588
**21**	0.738	0.721	0.721	0.574	0.531	0.345	0.721	0.721	0.431	0.711
**22**	0.659	0.644	0.644	0.721	0.630	0.510	0.850*	0.644	0.622	0.604
**23**	0.258	0.254	0.254	0.295	0.250	0.362	0.362	0.254	0.393	0.242
**24**	0.277	0.273	0.273	0.313	0.269	0.377	0.377	0.273	0.407	0.261
**25**	0.281	0.276	0.276	0.321	0.271	0.396	0.396	0.276	0.404	0.262
**26**	0.283	0.279	0.279	0.322	0.274	0.393	0.393	0.279	0.400	0.266
**27**	0.286	0.281	0.281	0.327	0.321	0.404	0.404	0.281	0.412	0.267
**28**	0.614	0.600	0.600	0.800	0.780	0.385	0.800	0.600	0.479	0.563

**29**	0.800	0.780	0.780	0.622	0.574	0.404	0.780	0.780	0.469	0.727
**30**	0.469	0.460	0.460	0.622	0.609	0.352	0.622	0.460	0.412	0.434
**31**	0.315	0.286	0.286	0.333	0.281	0.412	0.412	0.286	0.420	0.271
**32**	0.222	0.236	0.219	0.236	0.233	0.254	0.290	0.219	0.239	0.211
**33**	0.491	0.509	0.482	0.537	0.527	0.339	0.627	0.482	0.390	0.458
**34**	0.491	0.509	0.482	0.537	0.527	0.339	0.627	0.482	0.390	0.458
**35**	0.381	0.372	0.372	0.372	0.364	0.135	0.372	0.372	0.208	0.348
**36**	0.137	0.135	0.135	0.135	0.132	0.372	0.135	0.135	0.137	0.127
**37**	0.479	0.469	0.469	0.600	0.521	0.309	0.636	0.469	0.392	0.442
**38**	0.422	0.413	0.413	0.585	0.535	0.275	0.585	0.413	0.362	0.388
**39**	0.523	0.511	0.511	0.700	0.568	0.333	0.700	0.511	0.426	0.479
**40**	0.333	0.327	0.327	0.327	0.269	0.140	0.327	0.327	0.185	0.308

* The highest Tanimoto similarity value

Appendix 4. Mathematical Formulas Used to Calculate Pharmacophore Model Validation Parameters

$$\mathrm{TP}: \text{From ROC curve result}$$

$$\mathrm{FP}: \text{From ROC curve result}$$

$$\mathrm{FN} = \mathrm{N}_{\mathrm{active}} - \mathrm{TP}$$

$$\mathrm{TN} = \mathrm{N}_{\mathrm{decoy}} - \mathrm{FP}$$

$$\mathrm{PV} = \frac{\mathrm{N}_{\mathrm{active}}}{(\mathrm{N}_{\mathrm{active}} - \mathrm{N}_{\mathrm{decoy}})}$$

$$\mathrm{TPR} = \frac{\mathrm{TP}}{(\mathrm{TP}+\mathrm{FN})}$$

$$\mathrm{TNR} = \frac{\mathrm{TN}}{(\mathrm{TN}+\mathrm{FP})}$$

$$\mathrm{ACC} = \frac{(\mathrm{TP}+\mathrm{TN})}{(\mathrm{TP}+\mathrm{FN}+\mathrm{TN}+\mathrm{FP})}$$

$$\mathrm{PPV} = \frac{\mathrm{TP}}{(\mathrm{TP}+\mathrm{FP})}$$

$$\mathrm{NPV} = \frac{\mathrm{TN}}{(\mathrm{TN}+\mathrm{FN})}$$

$$\mathrm{FPR} = \frac{\mathrm{FP}}{(\mathrm{FP}+\mathrm{TN})}$$

$$\mathrm{FNR} = \frac{\mathrm{FN}}{(\mathrm{FN}+\mathrm{TP})}$$

$$\mathrm{MR} = \frac{(\mathrm{FN}+\mathrm{FP})}{(\mathrm{TP}+\mathrm{FN}+\mathrm{TN}+\mathrm{FP})}$$

$$\mathrm{GH} = \left(\frac{\mathrm{TP}\,(3\mathrm{N}_{\mathrm{active}}+\mathrm{TP}+\mathrm{FP})}{4\mathrm{N}_{\mathrm{active}}(\mathrm{TP}+\mathrm{FP})}\right)\left(1-\frac{\mathrm{FP}}{\mathrm{N}_{\mathrm{decoy}}}\right)$$

$$\mathrm{AUC}_{100\%} = \text{From ROC curve result}$$

$$\mathrm{EF}_{1\%} = \text{From ROC curve result}$$

Appendix 5. Validation Results of Pharmacophore Models for CXCR4 and CXCL12 Targets

Model	TP	FN	TN	FP	Validation Parameters*											
					PV	TPR	TNR	ACC	PPV	NPV	FPR	FNR	MR	GH	$AUC_{100\%}$	$EF_{1\%}$
**Internal Validation Target CXCR4**																
**1**	36	4	1015	985	0.02	0.90	0.51	0.52	0.04	1.00	0.49	0.10	0.48	0.13	0.83	10.2
**2**	36	4	1014	986	0.02	0.90	0.51	0.51	0.04	1.00	0.49	0.10	0.49	0.13	0.83	10.2
**3**	36	4	1015	985	0.02	0.90	0.51	0.52	0.04	1.00	0.49	0.10	0.48	0.13	0.83	10.2
**4**	36	4	1016	984	0.02	0.90	0.51	0.52	0.04	1.00	0.49	0.10	0.48	0.13	0.83	10.2
**5**	36	4	1016	984	0.02	0.90	0.51	0.52	0.04	1.00	0.49	0.10	0.48	0.13	0.83	10.2
**6**	36	4	1016	984	0.02	0.90	0.51	0.52	0.04	1.00	0.49	0.10	0.48	0.13	0.83	10.2
**7**	36	4	911	1089	0.02	0.90	0.46	0.46	0.03	1.00	0.54	0.10	0.54	0.11	0.80	10.2
**8**	36	4	1076	924	0.02	0.90	0.54	0.55	0.04	1.00	0.46	0.10	0.45	0.14	0.83	15.3
**9**	36	4	1084	916	0.02	0.90	0.54	0.55	0.04	1.00	0.46	0.10	0.45	0.14	0.83	12.8

Model	TP	FN	TN	FP	Validation Parameters*											
					PV	TPR	TNR	ACC	PPV	NPV	FPR	FNR	MR	GH	$AUC_{100\%}$	$EF_{1\%}$
**10**	36	4	1060	940	0.02	0.90	0.53	0.54	0.04	1.00	0.47	0.10	0.46	0.13	0.83	15.3
**External Validation Target CXCR4**																
**1**	8	2	211	289	0.02	0.80	0.42	0.43	0.03	0.99	0.58	0.20	0.57	0.09	0.78	10.2
**2**	8	2	212	288	0.02	0.80	0.42	0.43	0.03	0.99	0.58	0.20	0.57	0.09	0.78	10.2
**3**	8	2	211	289	0.02	0.80	0.42	0.43	0.03	0.99	0.58	0.20	0.57	0.09	0.78	10.2
**4**	8	2	211	289	0.02	0.80	0.42	0.43	0.03	0.99	0.58	0.20	0.57	0.09	0.78	10.2
**5**	8	2	211	289	0.02	0.80	0.42	0.43	0.03	0.99	0.58	0.20	0.57	0.09	0.78	10.2
**6**	8	2	211	289	0.02	0.80	0.42	0.43	0.03	0.99	0.58	0.20	0.57	0.09	0.78	10.2
**7**	8	2	180	320	0.02	0.80	0.36	0.37	0.02	0.99	0.64	0.20	0.63	0.08	0.71	10.2
**8**	8	2	215	285	0.02	0.80	0.43	0.44	0.03	0.99	0.57	0.20	0.56	0.09	0.71	10.2
**9**	8	2	217	283	0.02	0.80	0.43	0.44	0.03	0.99	0.57	0.20	0.56	0.10	0.71	10.2
**10**	8	2	213	287	0.02	0.80	0.43	0.43	0.03	0.99	0.57	0.20	0.57	0.09	0.71	10.2
**Internal Validation Target CXCL12**																
**1**	33	7	1949	51	0.02	0.83	0.97	0.97	0.39	1.00	0.03	0.18	0.03	0.49	0.91	48.4
**2**	33	7	1951	49	0.02	0.83	0.98	0.97	0.40	1.00	0.02	0.18	0.03	0.50	0.91	48.4
**3**	33	7	1951	49	0.02	0.83	0.98	0.97	0.40	1.00	0.02	0.18	0.03	0.50	0.91	51.0
**4**	33	7	1950	50	0.02	0.83	0.98	0.97	0.40	1.00	0.03	0.18	0.03	0.49	0.91	48.4
**5**	33	7	1951	49	0.02	0.83	0.98	0.97	0.40	1.00	0.02	0.18	0.03	0.50	0.91	48.4
**6**	33	7	1950	50	0.02	0.83	0.98	0.97	0.40	1.00	0.03	0.18	0.03	0.49	0.91	48.4
**7**	33	7	1951	49	0.02	0.83	0.98	0.97	0.40	1.00	0.02	0.18	0.03	0.50	0.91	48.4
**8**	33	7	1952	48	0.02	0.83	0.98	0.97	0.41	1.00	0.02	0.18	0.03	0.50	0.91	45.9
**9**	33	7	1954	46	0.02	0.83	0.98	0.97	0.42	1.00	0.02	0.18	0.03	0.51	0.91	45.9
**10**	33	7	1953	47	0.02	0.83	0.98	0.97	0.41	1.00	0.02	0.18	0.03	0.50	0.91	45.9
**External Validation Target CXCL12**																
**1**	10	0	482	18	0.02	1.00	0.96	0.96	0.36	1.00	0.04	0.00	0.04	0.50	1.00	51.0
**2**	10	0	482	18	0.02	1.00	0.96	0.96	0.36	1.00	0.04	0.00	0.04	0.50	1.00	51.0
**3**	10	0	482	18	0.02	1.00	0.96	0.96	0.36	1.00	0.04	0.00	0.04	0.50	1.00	51.0
**4**	10	0	482	18	0.02	1.00	0.96	0.96	0.36	1.00	0.04	0.00	0.04	0.50	1.00	51.0
**5**	10	0	482	18	0.02	1.00	0.96	0.96	0.36	1.00	0.04	0.00	0.04	0.50	1.00	51.0
**6**	10	0	481	19	0.02	1.00	0.96	0.96	0.34	1.00	0.04	0.00	0.04	0.49	1.00	51.0
**7**	10	0	482	18	0.02	1.00	0.96	0.96	0.36	1.00	0.04	0.00	0.04	0.50	1.00	51.0
**8**	10	0	484	16	0.02	1.00	0.97	0.97	0.38	1.00	0.03	0.00	0.03	0.52	1.00	51.0
**9**	10	0	483	17	0.02	1.00	0.97	0.97	0.37	1.00	0.03	0.00	0.03	0.51	1.00	51.0
**10**	10	0	484	16	0.02	1.00	0.97	0.97	0.38	1.00	0.03	0.00	0.03	0.52	1.00	51.0

*TP = True Positive; FN = False Negative; TN = True Negative; FP = False Positive; PV = Prevalence; TPR = True Positive Rate, TNR = True Negative Rate; ACC = Accuracy; PPV = Positive Predictive Value, NPV = Negative Predictive Value; FPR = False Positive Rate; FNR = False Negative Rate; MR = Misclassification Rate; GH = Goodness of Hit Score; $AUC_{100\%}$ = Area Under The ROC Curve 100%; $EF_{1\%}$ = Enrichment Factor 1%. The most reliable pharmacophore models are highlighted in grey.

Appendix 6. Receiver operating characteristic (ROC) curve of selected pharmacophore models. A, internal validation model 8 (target CXCR4); B, external validation model 8 (target CXCR4); C, internal validation model 3 (target CXCL12); D, external validation model 3 (target CXCL12)

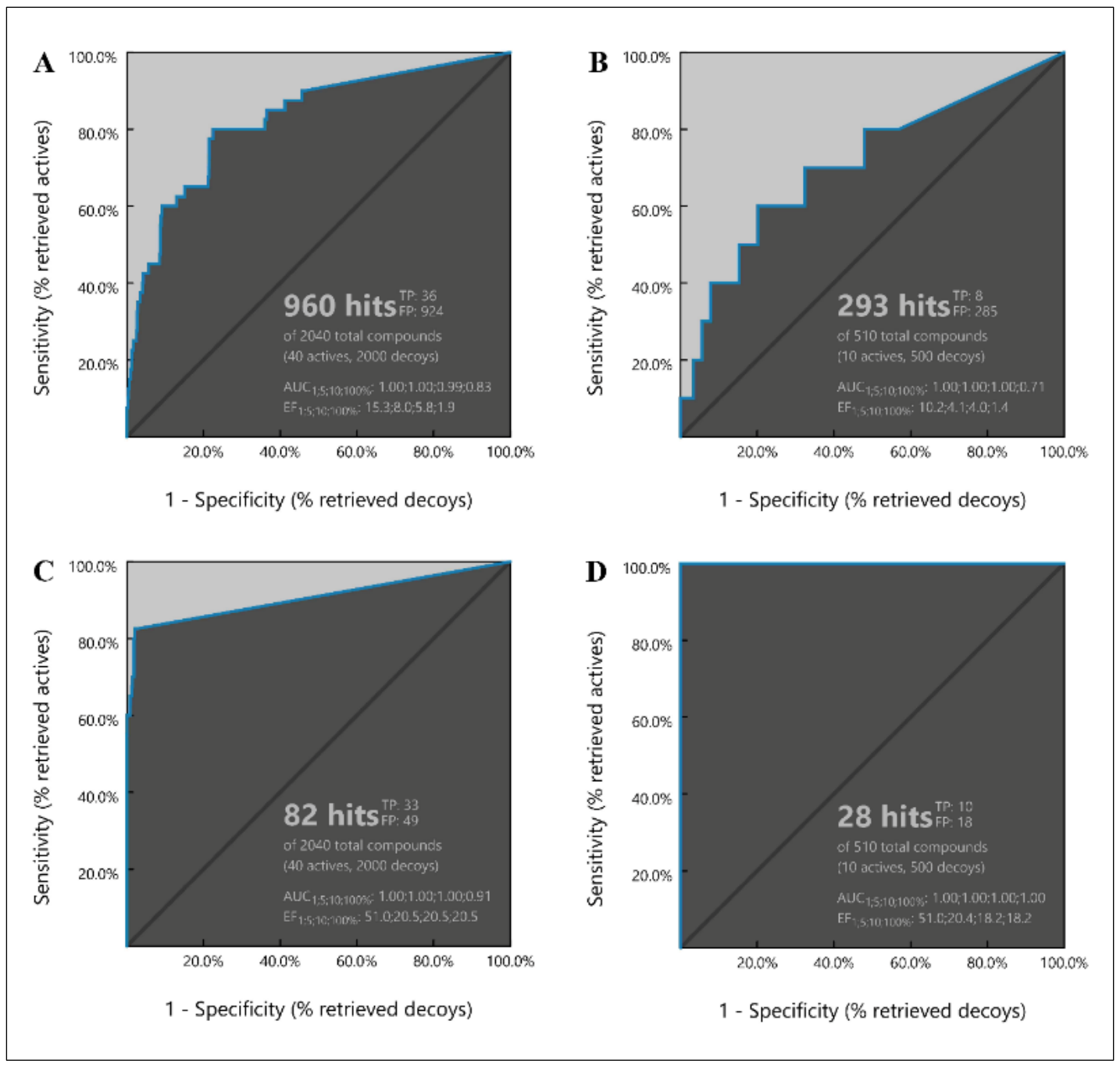

Appendix 7. Selected pharmacophore models. A, pharmacophore model 8 for CXCR4; B, pharmacophore model 3 for CXCL12

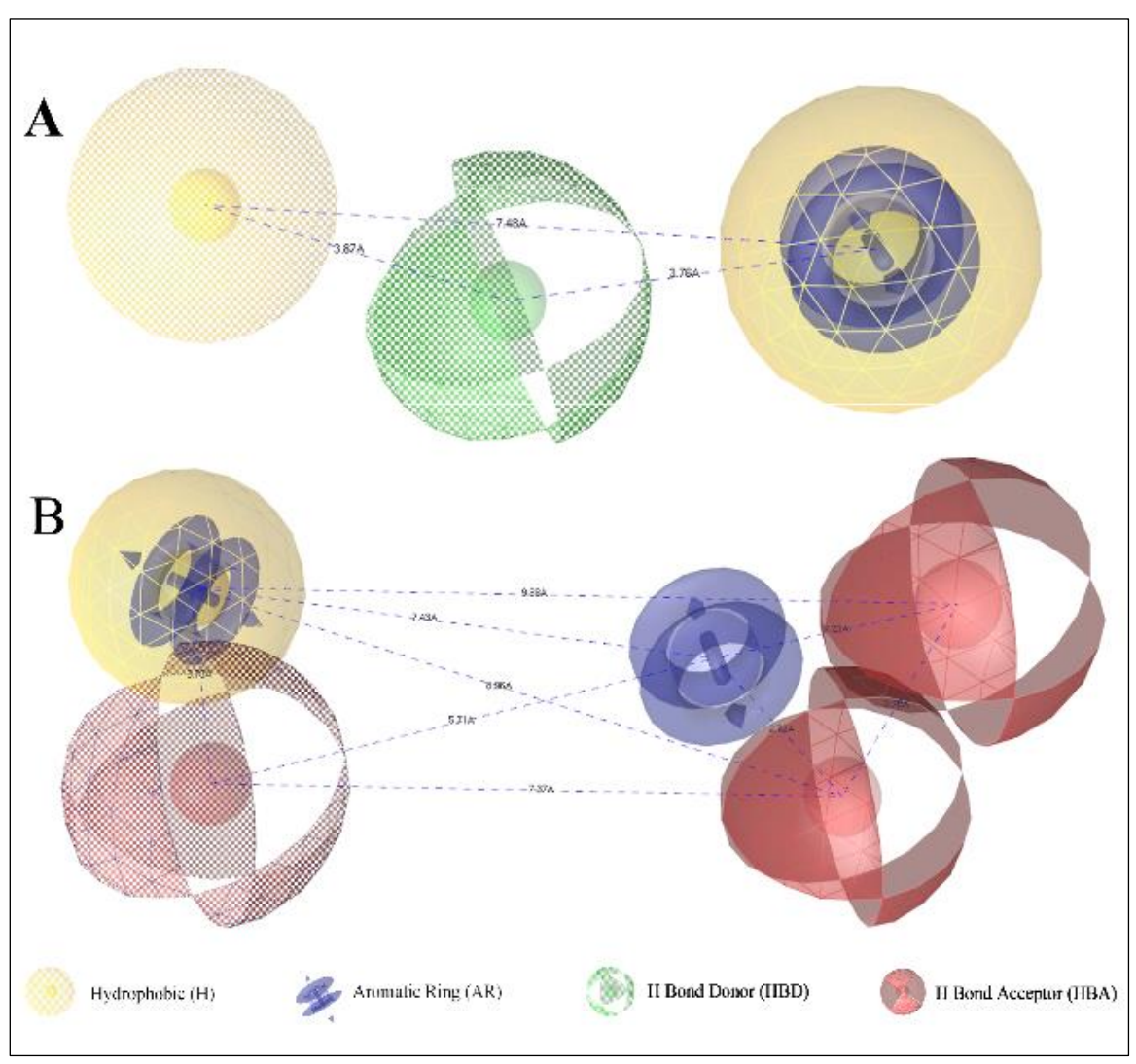

Appendix 8. Virtual Screening Results of Test Compounds with Selected Pharmacophore Models

No.	Compound	Pharmacophore-Fit Score	No.	Compound	Pharmacophore-Fit Score	No.	Compound	Pharmacophore-Fit Score
**Screening results of 79 test compounds and reference with pharmacophore model 8**								
**1**	D1	42.37	**22**	D28	34.64	**43**	D29	33.93
**2**	Mavorixafor	41.72	**23**	D73	34.59	**44**	D26	33.89

No.	Compound	Pharmacophore-Fit Score	No.	Compound	Pharmacophore-Fit Score	No.	Compound	Pharmacophore-Fit Score
**3**	D38	41.43	**24**	D25	34.54	**45**	D68	33.88
**4**	D10	35.97	**25**	D71	34.4	**46**	D33	33.84
**5**	D8	35.97	**26**	D32	34.39	**47**	D53	33.83
**6**	D7	35.97	**27**	D34	34.33	**48**	D46	33.81
**7**	D5	35.97	**28**	D35	34.33	**49**	D57	33.8
**8**	D3	35.94	**29**	D37	34.33	**50**	D78	33.75
**9**	D54	35.68	**30**	D40	34.33	**51**	D22	33.73
**10**	D42	35.54	**31**	D75	34.33	**52**	D44	33.62
**11**	D23	35.54	**32**	D76	34.33	**53**	D49	33.57
**12**	D9	35.52	**33**	D77	34.33	**54**	D36	33.49
**13**	D6	35.52	**34**	D78	34.33	**55**	D39	33.31
**14**	D2	35.52	**35**	D70	34.32	**56**	D51	33.26
**15**	D56	35.5	**36**	D65	34.19	**57**	D33	32.98
**16**	D24	35.5	**37**	D66	34.18	**58**	D36	32.98
**17**	D27	35.44	**38**	D48	34.14	**59**	D45	32.89
**18**	D41	35.05	**39**	D64	34.1	**60**	D50	32.85
**19**	D47	34.99	**40**	D31	34.01	**61**	D43	32.65
**20**	D79	34.75	**41**	D55	33.99			
**21**	D30	34.67	**42**	D79	33.97			
**Screening results of 79 test compounds and reference with pharmacophore model 3**								
**1**	D55	63.69	**7**	D54	63.52	**13**	D48	54.1
**2**	D44	63.65	**8**	D53	63.44	**14**	D46	54.1
**3**	D49	63.59	**9**	D45	63.39	**15**	D42	54.09
**4**	D56	63.59	**10**	LIT-927	55.62	**16**	D47	54.09
**5**	D50	63.55	**11**	D51	54.12	**17**	D41	54.08
**6**	D52	63.53	**12**	D43	54.1	**18**	D57	54.08

Appendix 9. Validation results of docking protocol. A, re-docking CXCR4-ITD; B, re-docking CXCL12-3GG

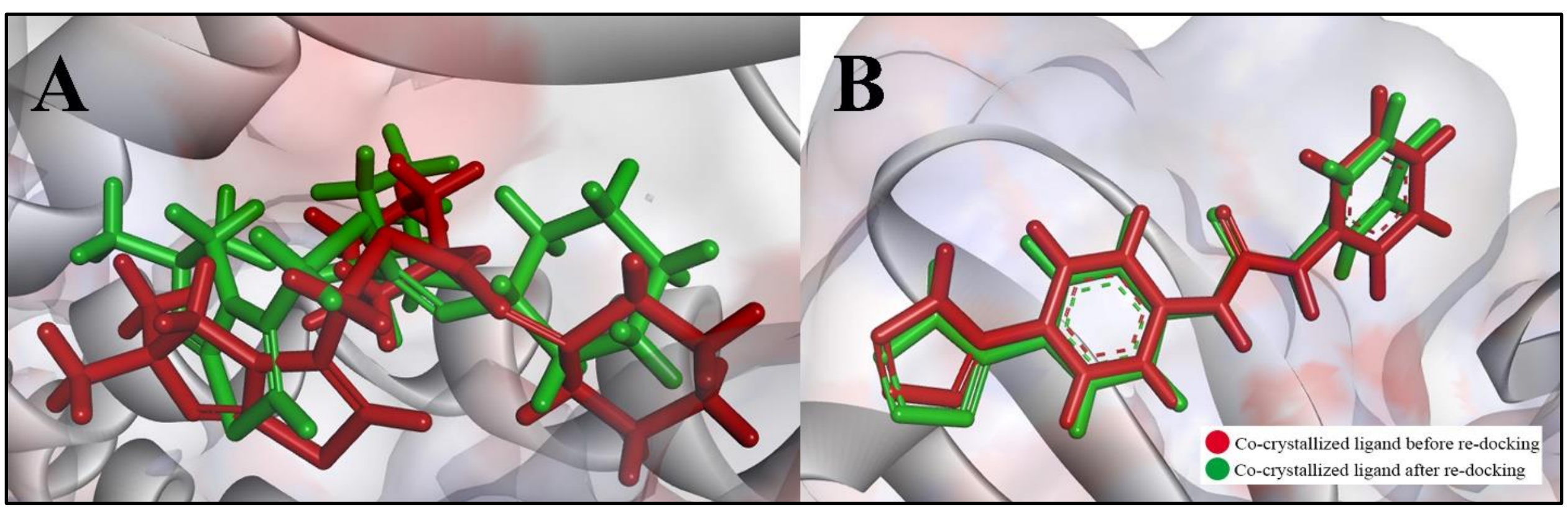

Appendix 10. Replicate-Specific Molecular Dynamics Simulation Parameters and MM-PBSA Results

Complex	MM-PBSA (kcal/mol)	MM-PBSA (Mean ± SD) (kcal/mol)	Mean RMSD (Å)	Mean RMSF (Å)	Mean Rg (Å)	Mean SASA (Å²)
CXCR4–mavorixafor (1)	-28.50	-32.39 ± 3.88	0.54 ± 0.05	0.18 ± 0.12	21.98 ± 0.15	1928.62 ± 40.72
CXCR4–mavorixafor (2)	-36.27		0.48 ± 0.04	0.19 ± 0.11	22.05 ± 0.22	1929.29 ± 48.03
CXCR4–D54 (1)	-29.11	-34.04 ± 4.93	0.53 ± 0.06	0.19 ± 0.15	22.34 ± 0.22	1943.44 ± 39.79
CXCR4–D54 (2)	-38.96		0.44 ± 0.04	0.21 ± 0.13	21.77 ± 0.31	1902.33 ± 37.57
CXCR4–D57 (1)	-47.69	-47.63 ± 0.06	0.50 ± 0.04	0.18 ± 0.10	22.19 ± 0.21	1931.39 ± 70.93
CXCR4–D57 (2)	-47.57		0.54 ± 0.04	0.15 ± 0.06	22.20 ± 0.12	1867.09 ± 41.90
CXCL12–LIT-927 (1)	-21.00	-16.86 ± 4.15	0.56 ± 0.10	0.29 ± 0.20	12.69 ± 0.28	565.23 ± 21.31
CXCL12–LIT-927 (2)	-12.71		0.56 ± 0.07	0.25 ± 0.18	12.41 ± 0.26	550.12 ± 23.06
CXCL12–D54 (1)	-19.65	-24.21 ± 4.56	0.46 ± 0.11	0.21 ± 0.18	12.68 ± 0.36	572.26 ± 17.28
CXCL12–D54 (2)	-28.77		0.47 ± 0.08	0.22 ± 0.17	12.82 ± 0.32	561.12 ± 19.27
CXCL12–D57 (1)	-23.60	-22.07 ± 1.53	0.48 ± 0.09	0.26 ± 0.21	13.58 ± 0.61	585.28 ± 23.04
CXCL12–D57 (2)	-20.54		0.52 ± 0.09	0.24 ± 0.19	12.83 ± 0.32	569.59 ± 17.59

Appendix 11. Hydrogen bond occupancy percentage of ligands in the CXCR4 and CXCL12 complexes

Complex	Donor	Acceptor	Occupancy (%)	Complex	Donor	Acceptor	Occupancy (%)
**CXCR4–mavorixafor (1)**	Lig-side	Glu288-Main	45.98	**CXCL12–LIT-927 (1)**	Lig-side	Glu15-side	14.46
	Lig-side	Ser285-main	29.64		Cys50-main	Lig-side	5.75
	Lig-side	Glu288-side	6.59		Cys11-side	Lig-side	1.20
	His113-side	Lig-side	5.16	**CXCL12–LIT-927 (2)**	Lig-side	Glu15-side	42.75
	Arg188-main	Lig-side	2.74		Cys50-main	Lig-side	30.57
	Arg188-side	Lig-side	2.30		Lig-main	Gln48-main	14.80
	Asp187-main	Lig-side	0.84		Arg47-side	Lig-main	8.81
**CXCR4–mavorixafor (2)**	Lig-side	Glu288-side	23.14		Gln48-main	Lig-side	2.11
	Lig-side	Trp94-side	21.03		Cys11-side	Lig-side	0.69
	Lig-side	Asp97-side	18.48		Val49-side	Lig-main	0.69
	Lig-side	His113-side	3.15		Arg47-side	Lig-side	0.60
	Lig-side	Asp187-side	1.95		Lig-side	Phe13-main	0.59
	Lig-side	Asp171-side	1.60	**CXCL12–D54 (1)**	Lys56-main	Lig-side	11.89
	His350-side	Lig-side	1.23		His17-main	Lig-main	5.05
	Ala291-main	Lig-side	1.04		Ala19-main	Lig-side	5.00
	Lig-side	Glu288-main	0.90		Trp57-side	Lig-side	5.00
	Leu91-main	Lig-side	0.71		Trp57-main	Lig-side	3.47
	His113-side	Lig-side	0.61		Lys56-side	Lig-side	3.39
	Phe292-side	Lig-side	0.61		Lig-side	His17-side	3.09
**CXCR4–D54 (1)**	Lig-side	Asp97-side	75.19		Lig-side	Ser16-main	1.57
	Tyr45-side	Lig-side	41.65		Arg20-main	Lig-side	1.50
	Lig-side	Trp94-main	29.40		Asn22-side	Lig-side	1.07

Complex	Donor	Acceptor	Occupancy (%)
	Asn37-side	Lig-side	2.88
	Lig-side	Asp97-main	2.28
	Ala98-main	Lig-side	1.96
	Trp94-side	Lig-side	1.86
	Ala98-side	Lig-side	1.65
	Lys38-main	Lig-side	1.44
	Leu41-side	Lig-side	1.27
	Lys38-side	Lig-side	1.05
	Pro42-side	Lig-side	0.52
**CXCR4–D54 (2)**	Lig-side	Asp97-side	64.13
	Asn37-side	Lig-side	24.94
	Lys38-side	Lig-side	15.40
	Lig-side	Trp94-main	11.50
	His281-side	Lig-side	6.84
	Lig-side	Tyr45-side	3.21
	Ala34-side	Lig-side	1.22
	His281-main	Lig-side	0.87
	Lig-side	Asp97-main	0.85
	Ser285-main	Lig-main	0.77
	Ala289-side	Lig-main	0.69
	Ser285-side	Lig-main	0.55
**CXCR4–D57 (1)**	Lig-side	Asp97-side	34.90
	Lig-side	Asp182-side	9.33
	Asn101-side	Lig-side	9.30
	Lys38-side	Lig-side	9.23
	Lig-side	Asp97-main	5.17
	Lys38-main	Lig-side	4.39
	Ala98-main	Lig-main	3.67
	Ala98-main	Lig-side	3.02
	Leu41-side	Lig-side	2.18
	Lig-side	Asp182-main	1.90
	Lig-side	Asn101-side	1.88
	Asn37-side	Lig-side	1.72
	Arg183-side	Lig-side	1.52
	Ala34-side	Lig-side	0.96
	Ala34-main	Lig-side	0.61
**CXCR4–D57 (2)**	Arg183-side	Lig-main	60.75
	Asn101-side	Lig-side	9.07
	Lig-side	Asp182-main	5.12
	Lys38-side	Lig-side	4.25
	Val99-main	Lig-side	2.05
	Ala98-main	Lig-side	1.45
	Lig-side	Tyr184-main	1.1
	Arg183-side	Lig-side	1.01
	Ala34-main	Lig-side	0.96
	His281-side	Lig-side	0.62
	Lys54-main	Lig-main	1.00
	Lig-side	Leu66-main	0.98
	Leu62-side	Lig-side	0.83
	Asn22-side	Lig-main	0.70
	Gln59-side	Lig-side	0.60
	Lig-side	Val18-main	0.59
	Gln37-side	Lig-main	0.56
**CXCL12–D54 (2)**	Lig-side	Ala35-main	28.28
	Gln59-side	Lig-main	13.92
	Gln37-main	Lig-side	7.13
	Lig-side	Gln37-side	2.90
	Lig-side	Pro53-main	2.74
	Gln37-side	Lig-side	1.67
	Gln59-side	Lig-side	1.48
	Ala19-main	Lig-side	1.47
	Asn67-main	Lig-main	1.12
	Asn22-side	Lig-main	0.85
	Pro53-side	Lig-side	0.84
	Leu62-side	Lig-side	0.80
	Asn67-side	Lig-side	0.77
	Asn44-side	Lig-side	0.61
	Lig-side	Gln59-side	0.58
**CXCL12–D57 (1)**	Lig-side	Glu63-side	11.32
	Lig-side	Phe14-main	7.54
	Phe14-main	Lig-side	7.41
	Cys9-main	Lig-main	5.58
	Cys9-main	Lig-side	5.46
	Lig-side	Glu15-side	1.97
	Lig-side	Leu66-main	1.77
	Ala35-main	Lig-side	1.29
	Cys34-main	Lig-side	1.13
	Lig-side	Asn33-main	0.92
	Lys64-main	Lig-side	0.92
	Gln59-main	Lig-main	0.91
	Arg8-main	Lig-side	0.89
	Ser4-main	Lig-side	0.75
	Arg8-main	Lig-main	0.74
	Lig-side	Leu5-main	0.7
	Gln59-side	Lig-main	0.59
**CXCL12–D57 (2)**	Lig-side	Glu15-side	35.08
	Cys50-main	Lig-main	20.28
	Lig-side	His17-main	5.40
	Val49-main	Lig-main	4.94
	Asn22-side	Lig-side	4.90
	Arg47-side	Lig-side	3.06
	Val18-main	Lig-side	1.51
	Ala19-main	Lig-main	1.16
	Cys50-main	Lig-side	1.10
	Ala19-main	Lig-side	1.00
	Lig-side	Asn45-side	0.93
	Cys11-side	Lig-main	0.93
	Val49-side	Lig-side	0.90
	Gln48-side	Lig-side	0.85
	Arg47-side	Lig-main	0.81
	Gln48-main	Lig-side	0.64
